# Exploring the interconnections between baseline symptoms in ultra-high risk youth who did and did not transition to psychosis over three years: A network analysis comparison

**DOI:** 10.1192/j.eurpsy.2025.10141

**Published:** 2025-11-27

**Authors:** Gabriele Lo Buglio, Simon Hartmann, Tommaso Boldrini, Scott R. Clark, Dominic Dwyer, Jessica A. Hartmann, Suzie Lavoie, Ashleigh Lin, Patrick D. McGorry, Josh Nguyen, Andrea Polari, Isabelle Scott, Annalisa Tanzilli, Andrew Thompson, Cassandra M.J. Wannan, Stephen J. Wood, Hok Pan Yuen, Alison R. Yung, Vittorio Lingiardi, Barnaby Nelson

**Affiliations:** 1 Department of Dynamic and Clinical Psychology, and Health Studies, Faculty of Medicine and Psychology, Sapienza University of Rome, Rome, Italy; 2 Department of Psychiatry, Faculty of Medicine, University of Ottawa, Ottawa, ON, Canada; 3 Ottawa Hospital Research Institute (OHRI), Ottawa, ON, Canada; 4 Discipline of Psychiatry, Adelaide Medical School, University of Adelaide, Adelaide, SA, Australia; 5 Centre for Youth Mental Health, University of Melbourne, Melbourne, VIC, Australia; 6 Orygen, Melbourne, VIC, Australia; 7 Department of Psychology and Educational Science, Pegaso Telematic University, Naples, Italy; 8 Department of Public Mental Health, Central Institute of Mental Health, Medical Faculty Mannheim, University of Heidelberg, Mannheim, Germany; 9 School of Population and Global Health, The University of Western Australia, Perth, WA, Australia; 10 Centre for Mental Health and Brain Sciences, Swinburne University of Technology, Australia; 11 School of Psychology, University of Birmingham, UK; 12 Institute for Mental and Physical Health and Clinical Translation, Deakin University, Geelong, VIC, Australia; 13 School of Health Sciences, University of Manchester, Manchester, M13 9PL, United Kingdom

**Keywords:** basic symptoms, early intervention, network analysis, positive symptoms, prevention, Ultra-high risk for psychosis

## Abstract

**Background:**

In the ultra-high risk for psychosis (UHR) field, it is unknown whether understanding symptom relationships, beyond symptom severity alone, may hold prognostic value and inform preventive care. In this study, network analysis was performed to examine the interconnections between baseline symptoms in UHR youth who did and did not transition to psychosis over three years.

**Methods:**

In a sample selected from the UHR1000+ cohort, positive and basic symptoms were assessed using the Comprehensive Assessment of At-Risk Mental States. Network analyses and network comparison tests were performed.

**Results:**

195 UHR youth transitioned to psychosis within three years and 346 did not. The two groups did not differ in the network structure, global strength (i.e., the overall level of connectivity between symptoms), or centrality of symptoms (i.e., their importance within networks). The transitioned group was characterized by unusual thought content not being connected to other symptoms; however, its centrality between networks was comparable. Across networks, impaired cognitive functioning connected disorganized speech to impaired emotional functioning, motor functioning, and tolerance to normal stress. Impaired bodily sensation connected perceptual abnormalities to other symptoms.

**Conclusions:**

The networks of youth who transitioned and who did not transition were similar, indicating similar baseline symptom relationships. Across groups, unusual thought content, despite being traditionally associated with transition, had little to no interactions with other symptoms. Clinical manifestations that may need attention include impaired cognitive functioning, which connected several symptoms, and impaired bodily sensation. Future research using time series data may support progress toward individualized care.

## Introduction

The ultra-high risk for psychosis (UHR) criteria have been developed to identify youth at increased risk of developing psychosis, enabling the provision of preventive treatments [1–3]. The UHR criteria focus on a combination of trait and state risk factors, with most cases exhibiting attenuated positive symptoms (e.g., unusual thought content and perceptual abnormalities) [4, 5].

The most important outcome in the field is the transition to psychosis [6], marking the point at which an individual has developed a full threshold or frank psychosis [4]. Research on differences between UHR youth who later develop psychosis and those who do not, focusing on factors associated with transition, is crucial to informing preventive care. While several studies have examined the prognostic value of the severity of symptoms [7, 8], growing international consensus emphasizes the need to refine prognostic models. To forecast psychosis onset, prediction models have been developed [4, 9, 10]; however, they have shown limited application in real-world clinical settings to date due to implementation gaps [11], suggesting the importance of novel, complementary approaches. An unexplored research area is whether focusing on the relationships among symptoms could differentiate youth who will develop psychosis from those who will not. Understanding symptom interconnections, beyond symptoms’ prevalence and/or severity alone, may hold a prognostic value and inform preventive strategies.

A promising approach to exploring the interconnections between symptoms is network analysis, which posits that symptoms should not be viewed as passive manifestations of a latent variable. Instead, symptoms are deemed to actively cause and reinforce each other within a complex system, driving the system toward an alternative, disordered stable state of prolonged symptom activation *–* what we recognize as “mental disorders” [12]. Symptom interactions can be visualized as a network, where some connections are stronger than others. Highly interconnected symptoms are considered important in sustaining and spreading psychopathology (centrality hypothesis) [13]. Network analysis represents a promising approach for framing diagnosis (i.e., identifying the interactions that sustain symptoms) and intervention (i.e., manipulating or modifying the interconnections between symptoms) [12]. Notably, within this approach, symptoms are understood as interconnected and interdependent, rather than isolated risk factors independently associated with transition.

Compared to more traditional approaches, network analysis may provide new perspectives on baseline differences between individuals who will transition and those who will not, potentially improving the detection of signs of a subsequent transition. For example, network analysis could clarify whether these groups differ in the pattern of associations between symptoms and how densely or strongly symptoms are connected [14]. At a more granular level, such an exploratory approach may suggest whether certain connections or symptoms have distinct roles within networks, potentially differing between groups. Moreover, understanding how symptoms are interconnected has the potential to identify symptoms that may need clinical attention and inform future studies.

Applying network analysis for this purpose shows promise, as it has differentiated clinical populations in previous studies, such as youth with a psychosis-risk state from clinical controls [15], and individuals with first-episode psychosis at baseline compared to 1-year follow-up [16]. Previous studies using network analysis in the field [15, 17–21] had reasonably modest sample sizes and did not compare symptom networks between individuals who later transitioned to psychosis and those who did not. Based on this background and utilizing data from the largest cohort with long-term follow-up in the field to date, this study aims to compare the baseline network structures of baseline symptoms in UHR individuals who transitioned to psychosis and those who did not within three years.

## Methods

### Participants and procedure

The UHR1000+ cohort comprised UHR youth who participated in studies conducted at Orygen, Melbourne, from 1995 to 2021 (Supplementary Table S1).

Given the wide variation in follow-up, which extended until 16.7 years, we established a three-year cut-off for transition. This cut-off was informed by a large-scale systematic review of psychosis prevention services, which explicitly recommends monitoring outcomes for at least three years, based on evidence that transition risk and clinical needs may extend beyond shorter-term follow-up periods [22]. Accordingly, we excluded youth who did not transition with less than three years of follow-up. Furthermore, UHR youth who developed psychosis after three years were categorized as having not transitioned to psychosis within three years in the main analysis and excluded from a sensitivity analysis (see “Network estimation”).

Participants were included if: a) they presented with a UHR state at baseline, and b) later developed psychosis or they were known to have not transitioned for at least three years. Exclusion criteria were: a) cases with missing follow-up date or missing symptom data to the extent that they could not be included in any correlation, b) a current or past psychotic disorder or manic episode; c) prior exposure to antipsychotic medication, with a total continuous haloperidol-equivalent dose exceeding 15–50 mg, depending on the original study; d) substance-induced psychotic disorder; e) a known medical condition that could explain symptoms; f) a diagnosis of a severe developmental disorder; g) a documented history of developmental delay or intellectual disability (IQ < 70); and h) inadequate English language proficiency for participants recruited in Australia.

Informed consent was obtained from all participants, as the studies included in the dataset were approved by the relevant local ethics committees. The authors assert that all procedures comply with the ethical standards of the relevant national and institutional committees on human research.

### Measures

UHR inclusion criteria were: trait and state risk factors (i.e., schizotypal disorder or a first-degree relative with a psychotic disorder plus functional decline or chronic low functioning), attenuated positive psychotic symptoms (APS) (i.e., attenuated positive symptoms), and brief limited intermittent psychotic symptoms (BLIPS) (i.e., short-lived psychotic episodes, remitting spontaneously within seven days). From 1999, functional decline or chronic low functioning was required for all UHR groups. From 1995 to 1996, UHR state was assessed using a combination of the Brief Psychiatric Rating Scale (BPRS) [23], the Comprehensive Assessment of Symptoms and History (CASH) [24], and the Global Assessment of Functioning (GAF) [25]. Between 1996 and 1999, the Comprehensive Assessment of At-Risk Mental States (CAARMS) [5] was used alongside the GAF. From 1999, the CAARMS, in conjunction with the Social and Occupational Functioning Assessment Scale [26] was employed.

Symptoms were assessed using different CAARMS versions. Due to their clinical relevance, complementary sets of clinical manifestations, and prognostic value [5, 27, 28], we selected baseline severity scores of positive symptoms (e.g., unusual thought content and perceptual abnormalities) and a range of psychopathology defined as basic symptoms in the CAARMS (e.g., subtle alterations of thoughts, behaviors, bodily perceptions, and affects [29]) described in Table 1. The harmonization process of the CAARMS items across different versions is described in Supplementary Table S2. The conversion procedure was derived from consensus among clinical experts in administering the CAARMS, optimizing comparability across different instrument versions. Harmonized CAARMS scores were used in previous publications [8, 30, 31].

**Table 1.** Description of symptoms considered in this study (edited and adapted from [5, 56])

Symptom	Symptom domain according to the CAARMS	Definition
Unusual thought content	Positive symptom	Referential thinking, thought control, insertion, mind reading, odd or eccentric ideas, perplexity, and delusional mood, such as the feeling that reality is subtly shifting or that something significant is about to unfold.
Non-bizarre ideas	Positive symptom	Fixed, plausible beliefs, including feelings of being persecuted, exaggerated self-opinion, ideas that the body is changing, guilt over past behaviors, nihilistic thoughts, excessive jealousy, intense religious or spiritual experiences, and erotomanic ideas.
Perceptual abnormalities	Positive symptom	Unusual sensory experiences, including visual, auditory, olfactory, gustatory, and tactile distortions or hallucinations, where youth perceive altered colors, sounds, smells, tastes, or sensations that feel real but may not really present.
Disorganized speech	Positive symptom	Difficulties in verbal communication, including problems finding the right words, using incorrect or irrelevant terms, going off-topic, being vague, repeating others’ words, or over-relying on gestures, which can make it challenging for listeners to follow the conversation.
Impaired cognitive functioning	Basic symptom	Subjectively reported difficulties with concentration, selective attention, thought coherence, comprehension, and memory (e.g., struggling to focus, easily becoming distracted), experiencing chaotic thoughts or thought blocking, misunderstanding others, or having memory lapses, particularly during stress.
Impaired emotional functioning	Basic symptom	Subjectively reported change in the ability to experience and express emotions, often manifesting as feelings of emotional emptiness (e.g., flattened facial expression, reduced eye contact, monotone speech), diminished pleasure in enjoyable activities, or a lack of typical emotional responses (e.g., inability to feel sadness during sad events). It also includes changes in affectivity that the person notices others have observed.
Impaired energy	Basic symptom	Subjectively reported marked reduction in physical and mental vitality, manifesting as feelings of exhaustion, reduced motivation, and physical weakness, which may impact daily activities, such as work or school and self-care. It also includes notions. It also includes whether the subject appears to take care of themselves (e.g., overall self-care)
Impaired motor functioning	Basic symptom	Subjectively reported difficulties with movement coordination, clumsiness, reduced spontaneity, and increased effort for routine actions. It may include unusual mannerisms or specific postures.
Impaired bodily sensation	Basic symptom	Subjectively reported unusual or unpleasant bodily sensations, like pulling, pain, itching, numbness, or abnormal sensations (e.g., vibrations), which may lead the person to perceive parts of their body as altered or different.
Impaired autonomic functioning	Basic symptom	Subjectively reported disruptions in autonomic functioning, such as irregular heart rate, rapid or deep breathing, nausea, sensitivity to weather changes, frequent urination, constipation, and sleep disturbances.
Impaired tolerance to normal stress	Basic symptom	Subjectively reported reduced ability to cope with everyday stressors, often resulting in increased irritability, discomfort, tension, or anxiety.

Regarding transition to psychosis, before 1999, it was identified using the BPRS/CASH and CAARMS. Since 1999, the CAARMS replaced the BPRS/CASH. In cases where CAARMS data were not available, public mental health records were reviewed.

### Statistical analyses

#### Group comparison

To compare baseline characteristics of UHR youth who developed psychosis with those who did not, continuous variables were analyzed using the Mann–Whitney U test due to deviations from normal distribution. Categorical variables were examined with the chi-square test, using Monte Carlo simulation (10,000 replicates) for cases with low expected frequencies. To maintain consistency with the approach used for comparing nodes/edges between networks (see “Network estimation”), symptoms’ *p*-values were adjusted using the Bonferroni correction. Youth who transitioned after three years were classified as not developing psychosis within the pre-defined three-year cut-off (main analysis) and were excluded in a secondary analysis.

### Network estimation

To avoid overlapping items, we used the goldbricker function of the networktools package [32], which identifies items that share similar relationships. The network structures were estimated using a Gaussian graphical model (GGM), which takes partial correlations as input, constructing the network by identifying pairwise statistical relationships (edges) between variables (nodes). An edge between two nodes indicates conditional dependence, with the edge weight reflecting the strength of this dependence. To estimate the network structures, the ggmModSelect algorithm, which has proven effective in consistently replicating individual edges [33], was employed. The algorithm generates multiple network structures by varying the LASSO tuning parameter. Then, the selected network is re-estimated using maximum likelihood estimation, optimizing a model selection criterion. Tuning was set to 0, corresponding to the Bayesian Information Criterion (BIC). Finally, the network is refined by iteratively adding or removing edges in a stepwise manner until the BIC criterion is optimized [33, 34]. To account for non-normal distribution, we used Spearman correlations [33]. Missing data were handled with pairwise deletion [35], in line with previous research [17].

For the main analysis, two network structures were built: (i) youth who transitioned within three years (ii) youth who did not transition within three years (including those who transitioned after three years). All symptoms in the networks were included if they had less than 5% of missing data. We assessed the centrality metric “strength,” which is the sum of the absolute values of all connection weights connected to a given node [36], reflecting the relative importance of nodes within the network. To compare the edges and centrality of nodes in each network structure, the edge weight difference test and the centrality difference test were performed, respectively.

To evaluate the stability of the networks, the accuracy of edge weights and the stability of centrality indices were assessed via bootstrap (Nboots = 2500). The correlation stability coefficient (CS) was examined, which indicates the maximum proportion of the sample that can be removed while maintaining a correlation of at least 0.7 between recalculated indices and those from the full sample. CS values above .25, .50, and .70 correspond to acceptable, good, and excellent stability, respectively [36]. The Network Comparison Test was used to compare the two network structures (youth who transitioned versus did not transition) in network structure, global strength (i.e., level of connectivity), and node strength [14]. In line with previous research [37], we presented edge weight differences only if differences in network structure and/or global strength were significant, and applied Bonferroni correction.

To test the robustness of our findings, we conducted sensitivity analyses. First, youth who developed psychosis after three years were excluded from the analysis (in contrast to the main analysis, in which they were part of the group that did not develop psychosis within our pre-defined three-year cut-off, as they transitioned later) and performed the Network Comparison Test. Second, we incorporated items with more than 5% of missing data into the main networks, performing the Network Comparison Test. Third, as sample size can affect network structures [33], a matched sub-sample of youth who did not transition, equal in size to the transitioned group, was generated. The groups were matched on age, sex assigned at birth, UHR inclusion criteria, and intervention provision (i.e., experimental versus placebo/enrollment in a cohort study), as they may influence baseline symptoms or transition [4, 38–40]. Then, this newly generated network structure was compared with that of youth who transitioned (main analysis) using the Network Comparison Test. Finally, to examine the impact of using different CAARMS versions, we estimated separate networks including transitioned and non-transitioned groups assessed using CAARMS versions with different scores (i.e., non-harmonized 0–4 and 0–6 scores were incorporated in separate networks).

Significance was set at *p* < .05. Analyses were performed using R version 4.3.1 [41] using the bootnet [36], qgraph [42], and NetworkComparisonTest [14] packages.

## Results

### Sample characteristics

Among the 1,242 UHR youth assessed for inclusion, 698 were excluded as they did not transition, and it was not known if they remained non-psychotic for at least three years. Three youth who transitioned were also excluded: two due to missing transition date and one due to missing data that prevented inclusion in any correlation. 541 youth were included: 195 transitioned within three years and 346 did not (Table 2). The mean duration between baseline assessment and transition was 276.72 days (SD = 267.33). The groups did not differ in age, sex assigned at birth, UHR inclusion criteria, or intervention provision. The transitioned group had greater impairment in global functioning, more severe general psychopathology, and experienced a longer duration between symptom onset and intake at clinical services. In this group, unusual thought content, impaired cognitive functioning, impaired energy, and impaired emotional functioning were more severe (Bonferroni-corrected). When excluding the 21 youth who transitioned after three years from the non-transitioned group, the observed pattern of results remained consistent (Supplementary Table S3).

**Table 2.** Socio-demographic and clinical characteristics at baseline of UHR youth who transitioned or did not transition to psychosis within three years

	Youth who did not transition	Youth who transitioned	*p*-value
Number of participants	346	195	
Age at baseline (mean ± SD)	18.51 (3.47)	18.80 (3.82)	0.442^a^
Gender assigned at birth (%)			
Male	153 (44.22)	92 (47.18)	0.566^b^
Female	193 (55.78)	103 (52.82)	
Time between first symptom and intake at clinical service, days (mean ± SD)	404.77 (778.66)	757.85 (1098.27)	<0.001^a^
GAF score (mean ± SD)	59.88 (10.71)	54.54 (11.12)	<0.001^a^
SOFAS score (mean ± SD)	54.85 (11.74)	51.83 (10.89)	0.050^a^
BPRS total score (mean ± SD)	45.38 (9.31)	47.78 (10.55)	0.042^a^
Unusual thought content severity score (mean ± SD)	3.02 (1.67)	3.51 (1.67)	0.004^a,1^
Perceptual abnormalities severity score (mean ± SD)	3.09 (1.72)	3.34 (1.69)	0.463^a,1^
Disorganized speech severity score (mean ± SD)	1.87 (1.43)	2.19 (1.40)	0.115^a,1^
Impaired cognitive functioning (mean ± SD)	2.35 (1.13)	2.63 (1.38)	0.031^a,1^
Impaired emotional functioning severity score (mean ± SD)	1.65 (1.44)	2.16 (1.68)	0.008^a,1^
Impaired energy severity score (mean ± SD)	2.61 (1.57)	3.02 (1.71)	0.029^a,1^
Impaired motor functioning severity score (mean ± SD)	0.55 (1.03)	0.80 (1.25)	0.360^a,1^
Impaired bodily sensation severity score (mean ± SD)	0.75 (1.26)	1.08 (1.55)	0.265^a,1^
Impaired autonomic functioning severity score (mean ± SD)	1.48 (1.54)	1.46 (1.63)	>0.999^a,1^
Impaired tolerance to normal stress (mean ± SD)	2.33 (1.66)	2.48 (1.85)	>0.999^a,1^
Non-bizarre ideas severity score (mean ± SD)	3.40 (1.83)	3.34 (1.56)	>0.999^a,1^
Time from baseline assessment to transition (mean ± SD)		276.72 (267.33)	
Time between baseline assessment and last follow-up or transition (if after three years) (mean ± SD)	2687.47 (1106.50)		
Enrolled in the context of (%)			
Cohort study/placebo	181 (52.31)	108 (55.38)	0.550^b^
Intervention treatment	165 (47.69)	87 (44.62)	
UHR inclusion criteria (%)			
BLIPS	12 (3.47)	13 (6.67)	0.109^b^
APS	226 (65.32)	131 (67.18)	
APS + BLIPS	15 (4.34)	8 (4.10)	
Trait	47 (13.58)	12 (6.15)	
BLIPS+Trait	2 (0.58)	2 (1.03)	
Trait+APS	40 (11.56)	24 (12.31)	
BLIPS+APS + Trait	4 (1.16)	4 (2.05)	
NA	0 (0.0)	1 (0.51)	

Abbreviations: APS, attenuated positive psychotic symptoms; BLIPS, brief limited intermittent psychotic symptoms; BPRS, Brief Psychiatric Rating Scale; GAF, Global Assessment of Functioning; NA, Not applicable; SOFAS, Social and Occupational Functioning Assessment Scale; UHR, ultra-high risk for psychosis; a, Mann–Whitney U test; b, *x*^2^ test/Monte Carlo; 1, after Bonferroni correction

### Network analysis

The goldbricker function did not detect nodes to be excluded. Due to nearly 60% missing data for the symptom “non-bizarre ideas” (Supplementary Table S4), this variable was excluded from the main analysis.

**Figure 1.**
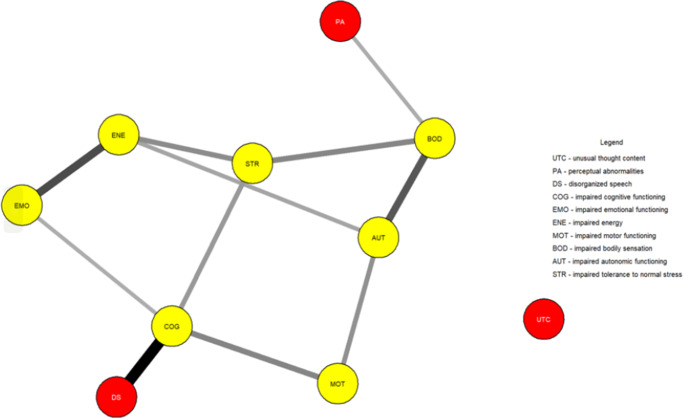
Network structure of youth who transitioned to psychosis (*N* = 195). The associations are either positive (colored black) or negative (colored red), with thicker lines representing stronger associations. Positive symptoms are shown as red nodes, while basic symptoms are shown as yellow nodes.

**Figure 2.**
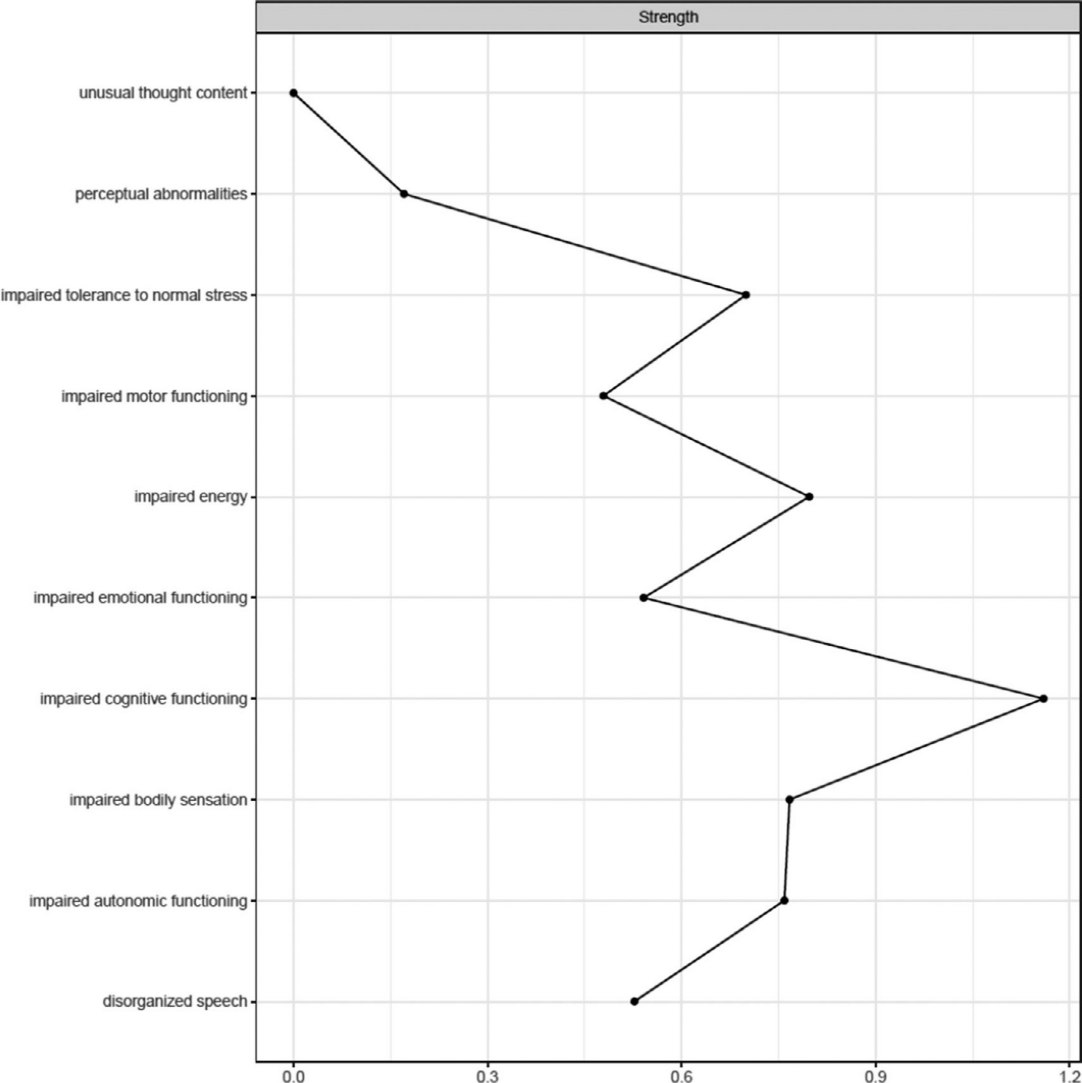
Centrality index (strength) of youth who transitioned to psychosis (*N* = 195), shown as standardized *z*-scores.

Figure 1 shows the network estimated from the youth who transitioned within three years. Unusual thought content was not connected to other symptoms, indicating that associations involving this symptom were too weak to be detected by the estimation method. Figure 2 plots the network centrality index strength. Impaired cognitive functioning, impaired energy, impaired bodily sensation, impaired autonomic functioning, and impaired tolerance to normal stress exhibited higher node strength than two positive symptoms (i.e., unusual thought content and perceptual abnormalities) (SupplementaryFigure S1). The edge between impaired cognitive functioning and disorganized speech was stronger than all other connections, except for those between impaired energy and emotional functioning, and between bodily sensation and autonomic functioning, where no difference was found (Supplementary Figure S2). The CS was 0.44 (Supplementary Figure S3). The bootstrapped 95% confidence intervals for the estimated edge weights are reported in Supplementary Figure S4.

**Figure 3.**
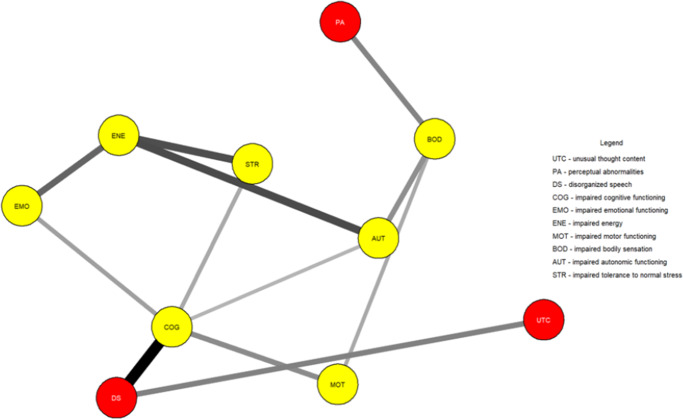
Network structure of youth who did not transition to psychosis (*N* = 346). The associations are either positive (colored black) or negative (colored red), with thicker lines representing stronger associations. Positive symptoms are shown as red nodes, while basic symptoms are shown as yellow nodes.

**Figure 4.**
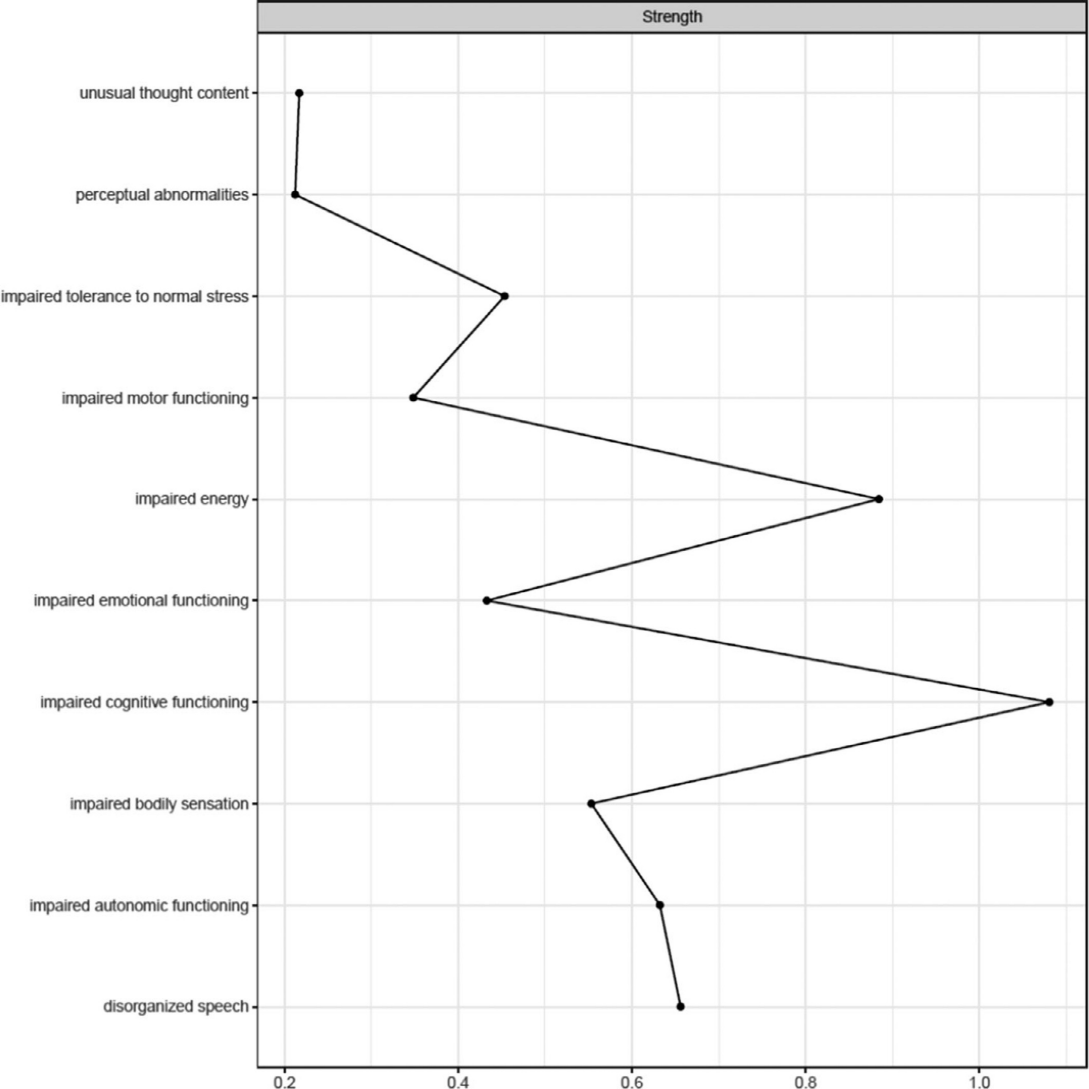
Centrality index (strength) of youth who did not transition (*N* = 346), shown as standardized z-scores.

Figure 3 shows the network estimated from youth who did not transition within three years. In these youths, in contrast to those who transitioned, unusual thought content exhibited a connection with disorganized speech. Figure 4 plots the network centrality indices. Impaired cognitive functioning and impaired energy exhibited higher node strength than two positive symptoms (i.e., perceptual abnormalities and unusual thought content) (Supplementary Figure S5). The edge between impaired cognitive functioning and disorganized speech was stronger than all the other connections in the network (Supplementary Figure S6). The CS was 0.44 (Supplementary Figure S7). The bootstrapped 95% confidence intervals for the estimated edge weights are reported in Supplementary Figure S8.

In both networks, impaired cognitive functioning connected disorganized speech to impaired emotional functioning, impaired motor functioning, and impaired tolerance to normal stress. Impaired bodily sensation connected perceptual abnormalities to other symptoms.

The Network Comparison Test revealed that the two network structures did not differ in global strength (*p* = .53), network structure (*p* = .42), and node strength (*p* > .05 in all nodes) (Supplementary Table S5).

Sensitivity analyses confirmed the lack of significant differences in the two groups and the isolation of unusual thought content in youth who transitioned. This pattern of results was observed when excluding youth who transitioned after three years from the group of youth who did not transition within three years (Supplementary Figures S9–S12, Supplementary Table S6). Such results were unchanged when incorporating “non-bizarre ideas” in the two main networks (Supplementary Figures S13–S20, Supplementary Table S7) and when comparing youth who transitioned (main analysis) with a sub-sample of youth who did not transition matched for sample size, age, sex assigned at birth, UHR inclusion criteria, and intervention provision (Supplementary Figures S21–S24, Supplementary Table S8). Networks estimated from the transitioned and non-transitioned groups assessed using CAARMS versions with different scores were similar but unstable, not allowing us to draw conclusions regarding the impact of different CAARMS versions on symptom interconnections (Supplementary Figures S25–S32, Supplementary Tables S9–S10).

Finally, we compared excluded and included youth who did not transition (main analysis), observing baseline differences, including some symptoms’ severity and network structure. However, only differences in one edge remained significant after Bonferroni correction (Supplementary Tables S11–S12, Supplementary Figures S33–S34).

## Discussion

In 541 UHR young people recruited over more than 25 years, we examined the interconnections between baseline symptoms in youth who transitioned and those who did not within three years from assessment.

Results showed that network analysis of baseline symptoms did not differentiate between youth who transitioned from those who did not. The two groups exhibited a comparable level of connectivity, symptom interconnections, and the importance of individual symptoms within networks. In contrast, differences in symptoms’ severity were observed, namely unusual thought content, impaired cognitive functioning, impaired emotional functioning, and impaired energy.

The most notable difference was unusual thought content being isolated in the transitioned group. This indicates that such a symptom was not meaningfully connected to other symptoms within the constraints of the estimation procedure. We could hypothesize that the isolation of this symptom could reflect its tapping into unusual experiences related to a disruption of the barrier between the self and the world–phenomena traditionally seen as part of the Schneiderian first-rank symptoms and distinct from other positive symptoms and clinical manifestations [43, 44]. However, its strength did not differ between groups, indicating comparable importance across networks. Although unusual thought content represents one of the most robust risk factors for transition [45], potentially leading to a loss of insight and diminished contact with reality, it had little to no interaction with other symptoms across networks. Moreover, although positive symptoms define two of the three UHR subgroups and mark transition, most of them had little importance within networks, in line with prior studies [15, 21]. Symptoms like impaired cognitive functioning and impaired energy exhibited several interconnections; however, limited research has explored the prognostic value of highly interconnected symptoms, with mixed findings [46]. Our sensitivity analyses supported the comparability of network structures and the isolation of unusual thought content in those who transitioned.

Across groups, network analysis revealed a complex interplay between speech, perceptual, cognitive, affective, and bodily symptoms. Findings suggest clinical attention for impaired cognitive functioning, as it is linked to disorganized speech to emotional, bodily, and stress-related impairments. A strong connection was between impaired cognitive functioning and disorganized speech, suggesting a shared underlying problem related to disorganized thinking. The results also suggest the potential role of impaired bodily sensation in linking perceptual abnormalities to other symptoms.

According to network comparisons, aside from the isolation of unusual thought content in the transitioned group *–* which requires further research before clinical translation *–* no symptom emerged as warranting clinical attention specifically in those who later transitioned. Research to inform more effective treatments is justified, as emphasized by an updated meta-analysis, which highlighted no sustained effects of any intervention in preventing transition compared to control conditions [47]. In that study, the most promising psychosocial intervention was the integrated approach by Bechdolf et al. (i.e., cognitive-behavioral therapy, cognitive remediation, skills training, and psychoeducation) [48], which might target multiple symptoms in our networks (e.g., impaired cognitive functioning and impaired tolerance to normal stress, also managing thought and perceptual alterations); however, it was trialed in individuals only with basic symptoms (without subthreshold psychotic symptoms). Our results suggest clinical attention on positive symptoms alongside a range of psychopathology, as symptoms like impaired cognitive functioning and impaired tolerance to normal stress connected multiple clinical manifestations.

Cross-sectional networks may fail to capture the complex, dynamic change process that marks the transition from one state of the system of symptoms (e.g., UHR) to another (e.g., psychosis), in which such a system reaches a critical, tipping point and remains trapped in a novel equilibrium [12, 49]. To refine prognostic precision, studies using network approaches to analyze panel or time series data hold promise; for a framework, see [50]. These models estimate how symptoms influence one another over time by capturing lagged, directional interactions *–* potentially enhancing prognostic accuracy [51, 52]. Notably, this complies with ongoing international research programs that perform repeated assessments in UHR youth [9]. Such research could explore how positive symptoms reinforce each other and/or are sustained by other symptoms over time, potentially contributing to the progression toward psychosis.

## Limitations

Several limitations of the current study should be acknowledged. First, the three-year follow-up needs to be fully incorporated into preventive care. However, monitoring outcomes over this period complies with large-scale evidence synthesis studies on psychosis prevention services and transition [22, 53]. Second, although the CAARMS includes items related to basic symptoms, it mainly focuses on UHR identification, differing from tools assessing basic symptoms risk inclusion criteria [54, 55]. However, by harmonizing different CAARMS versions, we could adopt a homogeneous approach across heterogeneous studies. Third, excluded and included youth who did not transition exhibited baseline differences. However, only one connection remained significant after the Bonferroni correction. Finally, different CAARMS versions and tools (e.g., BPRS, GAF, CASH) were used to assess symptoms and UHR status. A comprehensive CAARMS symptoms harmonization was guided by clinical anchor descriptions to preserve the meaning of severity ratings and was developed through expert consensus, increasing statistical power. We performed sensitivity analyses to investigate the impact of using different CAARMS versions, resulting in unstable networks.

## Conclusion

The networks of youth who transitioned and who did not transition were similar, indicating similar baseline symptom relationships. Across groups, unusual thought content, despite being traditionally associated with transition, had little to no interactions with other symptoms. Clinical manifestations that may need attention include impaired cognitive functioning and impaired bodily sensation. Future research using time series data may support progress toward individualized care.

## Supporting information

10.1192/j.eurpsy.2025.10141.sm001 Lo Buglio et al. supplementary material Lo Buglio et al. supplementary material

## Data Availability

Data are available upon request from the corresponding author.

## Supplementary material

The supplementary material for this article can be found at http://doi.org/10.1192/j.eurpsy.2025.10141.
