# Supplementary material for: Exploring the interconnections between baseline symptoms in ultra-high risk youth who did and did not transition to psychosis over three years: A network analysis comparison
Source: Eur Psychiatry. 2025 Nov 27;69(1):e7. doi: 10.1192/j.eurpsy.2025.10141 (PMC12835566; doi:10.1192/j.eurpsy.2025.10141)
Supplement: Lo Buglio et al. supplementary material [file S0924933825101417sup001.docx]

**Supplementary material to**

**Exploring the interconnections between baseline symptoms in ultra-high risk youth who did and did not transition to psychosis over three years: A network analysis comparison**

**Table of contents**

**Table S1.** Studies included in the dataset and participants included in this study p.5

**Table S2.** Harmonization of CAARMS items p.6

**Table S3.** Socio-demographic and clinical characteristics at baseline of individuals who transitioned or did not transition to psychosis within three years, excluding those who transitioned after three years p. 10

**Table S4.** Proportion of missing data for each variable p. 12

**Figure S1.** Centrality difference test in the network of individuals who transitioned p.13

**Figure S2.** Edge weight difference test in the network of individuals who transitioned p.14

**Figure S3.** Average correlation between the centrality indices of the network subsamples and the original sample in the network of individuals who transitioned p.15

**Figure S4.** Bootstrapped confidence intervals of estimated edge weights for the network of individuals who transitioned p.16

**Figure S5.** Centrality difference test in the network of individuals who did not transition p.17

**Figure S6.** Edge weight difference test in the network of individuals who did not transition p.18

**Figure S7.** Average correlation between the centrality indices of the network subsamples and the original sample in the network of individuals who did not transition p.19

**Figure S8.** Bootstrapped confidence intervals of estimated edge weights for the network of individuals who did not transition p.20

**TableS5.** Network comparison test to compare the network of individuals who transitioned and who did not transition p.21

**Figure S9.** Network structure of individuals who did not transition excluding those who transitioned after three years p. 22

**Figure S10.** Centrality index (strength) in individuals who did not transition excluding those who transitioned after three years p. 23

**Figure S11.** Average correlation between the centrality indices of the network subsamples and the original sample in individuals who did not transition excluding those who transitioned after three years p. 24

**Figure S12.** Bootstrapped confidence intervals of estimated edge weights for the network of individuals who did not transition within three years excluding those who transitioned after three years p.25

**TableS6.** Network comparison test to compare the network structures of individuals who transitioned and who did not transition within three years, excluding those who transitioned after three years p.26

**Figure S13.** Network structure of individuals who transitioned including the node “non-bizarre ideas” p. 27

**Figure S14.** Centrality index (strength) in the network of individuals who transitioned including “non-bizarre ideas” p.28

**Figure S15.** Average correlation between the centrality indices of the network subsamples and the original sample in the network of individuals who transitioned including “non bizarre ideas” p.29

**Figure S16.** Bootstrapped confidence intervals of estimated edge weights for the network of individuals who transitioned including “non-bizarre ideas” p.30

**Figure S17.** Network structure of individuals who did not transition including the node “non-bizarre ideas” p. 31

**Figure S18.** Centrality index (strength) in the network of individuals who did not transition including “non-bizarre ideas” p.32

**Figure S19.** Average correlation between the centrality indices of the network subsamples and the original sample in the network of individuals who did not transition including “non bizarre ideas” p.33

**Figure S20.** Bootstrapped confidence intervals of estimated edge weights for the network of individuals who did not transition including “non-bizarre ideas” p.34

**TableS7.** Network comparison test to compare the network of individuals who transitioned and who did not transition, both including “non-bizarre ideas” p.35

**Figure S21.** Network structure of the matched sub-group of individuals who did not transition p.36

**Figure S22.** Centrality index (strength) in the matched sub-group of individuals who did not transition p.37

**Figure S23.** Average correlation between the centrality indices of the network subsamples and the original sample in the network of the matched sub-group of individuals who did not transition p.38

**Figure S24.** Bootstrapped confidence intervals of estimated edge weights for the network of the matched sub-group of individuals who did not transition p.39

**TableS8.** Network comparison test to compare the network of individuals transitioning and matched sub-group of individuals who did not transition p.40

**Figure S25.** Network structure of individuals who transitioned using non-harmonized CAARMS scores (0-4) p. 41

**Figure S26.** Average correlation between the centrality indices of the network subsamples and the original sample in the network of individuals who transitioned using non-harmonized CAARMS versions (0-4 scores) p. 42

**Figure S27.** Network structure of individuals who did not transition using non-harmonized CAARMS versions (0-4 scores) p. 43

**Figure S28.** Average correlation between the centrality indices of the network subsamples and the original sample in the network of individuals who did not transition using non-harmonized CAARMS versions (0-4 scores) p. 44

**TableS9.** Network comparison test to compare the network of individuals transitioning and individuals who did not transition using non-harmonized CAARMS versions (0-4 scores) p. 45

**Figure S29.** Network structure of individuals who transitioned using non-harmonized CAARMS versions (0-6 scores) p. 46

**Figure S30.** Average correlation between the centrality indices of the network subsamples and the original sample in the network of individuals who transitioned using non-harmonized CAARMS versions (0-6 scores) p. 47

**Figure S31.** Network structure of individuals who did not transition using non-harmonized CAARMS versions (0-6 scores) p. 48

**Figure S32.** Average correlation between the centrality indices of the network subsamples and the original sample in the network of individuals who did not transition using non-harmonized CAARMS versions (0-6 scores) p. 49

**TableS10.** Network comparison test to compare the network of individuals transitioning and individuals who did not transition using non-harmonized CAARMS versions (0-6 scores) p. 50

**Table S11.** Socio-demographic and clinical characteristics at baseline of excluded individuals and included individuals did not transition to psychosis within three years p.51

**Figure S33.** Network structure of excluded individuals who did not transition p.53

**Figure S34.** Average correlation between the centrality indices of the network subsamples and the original sample in the network of excluded individuals who did not transition p.54

**Table S12.** Network comparison test to compare the network of excluded individuals and included individuals who did not transition to psychosis within three years p. 55

**Table S1.** Studies included in the dataset and participants included in this study

These studies were mainly conducted at the Personal Assessment and Crisis Evaluation (PACE) clinic, the Early Psychosis Prevention and Intervention Centre (EPPIC), and four headspace centers (i.e., youth mental health services [1]). This cohort also included data of participants from countries other than Australia, recruited in an international multi-centre trial [2]. Details on the included studies are reported elsewhere [3].

| Study | Recruitment years | Age at baseline | N participants (N transitioned within three years) |
| --- | --- | --- | --- |
| Yung et al. [4] | 1995-1996 | 14-30 years | 41 (23) |
| McGorry et al. [5] | 1996-1999 | 14-30 years | 82 (31) |
| Thompson et al. [6] | 2000 | 14-30 years | 34 (8) |
| Berger et al. [7] | 2000-2005 | 14-30 years | 186 (30) |
| Yung et al. [8] | 2000-2007 | 14-30 years |  |
| Phillips et al. [9] | 2000-2005 | 14-30 years |  |
| Nelson et al. [10] | 2008-2010 | 15-25 years | 13 (13) |
| Amminger et al.[2]  McGorry et al. [11] | 2010-2014 | 13-40 years | 133 (39) |
| EU-GEI [12] | 2012-2015 | 15-35 years | 6 (6) |
| Nelson et al. [13] | 2014-2018 | 15-25 years | 2 (2) |
| Hartmann et al. [14] | 2016-2018 | 12-25 years | 2 (2) |
| Nelson et al. [15] | 2016-2019 | 12-25 years | 37 (36) |
| Tognin et al. [16] | 2017-2019 | 15-25 years | 2 (2) |
| Bayer et al. [17] | 2018-2021 | 12-25 years | 3 (3) |

**Table S2.** Harmonization of CAARMS items

In the UHR 1000, the severity, frequency, onset date, and offset date are recorded for the four positive symptoms (unusual thought content, non-bizarre ideas, perceptual abnormalities, and disorganized speech). For all other items, the severity score was recorded. CAARMS measures for studies prior to 2006 were converted to the new CAARMS scale introduced in 2006 (see conversion guideline below). Scale is 0-6.

In this study, we selected positive and basic symptoms. For item pairs that focusing on the same symptom, we retained the positive symptom.

| Domain  (Old CAARMS domains) | Old CAARMS (Used in Prediction Study, First Intervention Study, and Stress Cortisol Study) | New CAARMS 2002 version (Used in Lithium trial, Ris-Aus-9, and Monitoring) | New CAARMS 2006 version (Used in studies later than 2005) | Old CAARMS Conviction scale (0-4) converted into Score on CAARMS 2006 intensity scale (0-6) |
| --- | --- | --- | --- | --- |
| Unusual Thought Content (UTC) | 1.1 Disorders of thought content – take the highest score (0-4) across the conviction scales of ‘obsessions’, ‘pre-delusional beliefs’, ‘non-bizarre ideas’, ‘bizarre ideas’ | 1.1 Disorders of thought content | 1.1 Unusual thought content | 0 = 0  1 = 1  2 = 3  3 = 5  4 = 6 |
| Non-Bizarre Ideas (NBI) | NA | NA | 1.2 Non-bizarre Ideas |  |
| Perceptual Abnormalities (PA) | 2.1 Perceptual abnormalities – take the highest score (0-4) across the presence scales of visual, auditory, olfactory, gustatory, somatic, tactile illusions and hallucinations | 1.2 Perceptual abnormalities | 1.3 Perceptual abnormalities | 0 = 0  1 = 1  2 = 2  3 = 4  4 = 5 |
| Disorganised Speech (DS) | 3.1.1 Conceptual disorganization – take the highest score (0-4) across the intensity scales of perceived abnormalities, comprehension difficulties, awareness of others’ difficulties objective assessment of speech abnormalities, and observed abnormalities (3.1.2) | 1.3 Disorganised speech | 1.4 Disorganised speech | 0 = 0  1 = 1  2 = 2  3 = 4  4 = 6 |
| Motor Disturbances | 4.1 Intensity of motor disturbance | 6.1 and 6.2 Take the higher score of subjective complaints of impaired motor functioning and observed changes in motor functioning | 6.1 and 6.2 Take the higher score of subjective complaints of impaired motor functioning and observed changes in motor functioning | 0 = 0  1 = 1  2 = 3  3 = 5  4 = 6 |
| Disorders of Concentration, Attention and Memory | 5.1.1 Disorders of concentration and attention – the intensity of concentration and attention disturbance score (0-4) | 2.1 and 2.2 Take the higher score of subjective cognitive change and observed cognitive change | 2.1 and 2.2 Take the higher score of subjective cognitive change and observed cognitive change | 0 = 0  1 = 1  2 = 2  3 = 4  4 = 6 |
| Disorders of Emotion and Affect | 6.1.1 and 6.2.1 Take the higher score of impaired emotional functioning intensity and change in affect intensity | 3.1 and 3.2 Take the higher score of subjective emotional disturbance and observed blunted affect | 3.1 and 3.2 Take the higher score of subjective emotional disturbance and observed blunted affect | 0 = 0  1 = 1  2 = 2  3 = 4  4 = 6 |
| Subjectively Impaired Energy | 7.1 Subjective complaints of impaired energy | 4.2 Avoliton/apathy | 4.2 Avoliton/apathy | 0 = 0  1 = 1  2 = 3  3 = 5  4 = 6 |
| Impaired tolerance to normal stress | 8.1 Subjective complaints of impaired tolerance to normal stress | 7.8 Impaired tolerance to normal stress | 7.8 Impaired tolerance to normal stress | 0 = 0  1 = 1  2 = 3  3 = 5  4 = 6 |
| “Basic symptoms”  The Old CAARMS already contains scales for the basic symptoms (see second column). The corresponding scales in the CAARMS Jan 2002 & 2006 version are listed in the third and fourth column. The scoring system for matching them up appears in the right-hand column. | | | | |
| Impaired cognitive functioning | Subjective complaints of impaired cognitive functioning | 2.1 Subjective experience of change in attention/concentration | 2.1 Subjective experience of change in attention/concentration | 0 = 0  1 = 1  2 = 3  3 = 5  4 = 6 |
| Impaired emotional functioning | Subjective complaint of impaired emotional functioning | 3.1 Subjective emotional disturbance | 3.1 Subjective emotional disturbance | 0 = 0  1 = 1  2 = 3  3 = 5  4 = 6 |
| Impaired energy | Subjective complaints of impaired energy | 4.2 Avoliton/apathy | 4.2 Avoliton/apathy | 0 = 0  1 = 1  2 = 3  3 = 5  4 = 6 |
| Impaired motor functioning | Subjective complaints of impaired motor functioning | 6.1 Subjective complaints of impaired motor functioning | 6.1 Subjective complaints of impaired motor functioning | 0 = 0  1 = 1  2 = 3  3 = 5  4 = 6 |
| Impaired bodily sensation | Subjective complaints of impairment of bodily sensation | 6.3 Subjective complaints of impaired bodily sensation | 6.3 Subjective complaints of impaired bodily sensation | 0 = 0  1 = 1  2 = 3  3 = 5  4 = 6 |
| Impaired external perception | Subjective complaints of impaired external perception | 1.2 Perceptual abnormalities | 1.2 Perceptual abnormalities | 0 = 0  1 = 1  2 = 3  3 = 4  4 = 6 |
| Impaired autonomic functioning | Subjective complaints of impaired autonomic functioning | 6.4 Subjective complaints of impaired autonomic functioning | 6.4 Subjective complaints of impaired autonomic functioning | 0 = 0  1 = 1  2 = 3  3 = 5  4 = 6 |
| Impaired tolerance to normal stress | Subjective complaints of impaired tolerance to normal stress | 7.8 Impaired tolerance to normal stress | 7.8 Impaired tolerance to normal stress | 0 = 0  1 = 1  2 = 3  3 = 5  4 = 6 |

**Table S3.** Socio-demographic and clinical characteristics at baseline of individuals who transitioned or did not transition to psychosis within three years, excluding those who transitioned after three years from the former group

|  | Individuals who did not transition | Individuals who transitioned | p |
| --- | --- | --- | --- |
| Number of participants | 325 | 195 |  |
| Age at baseline (mean ± SD) | 18.45 (3.48) | 18.80 (3.82) | 0.318^a^ |
| Gender assigned at birth (%) |  |  |  |
| Male | 143 (44.00) | 92 (47.18) | 0.539^b^ |
| Female | 182 (56.00) | 103 (52.82) |  |
| Time between first symptom and intake at clinical service, days (mean ± SD) | 383.00 (678.45) | 757.85 (1098.27) | <.001^a^ |
| GAF score (mean ± SD) | 60.20 (10.76) | 54.54 (11.12) | <.001^a^ |
| SOFAS score (mean ± SD) | 54.99 (11.74) | 51.83 (10.89) | 0.043^a^ |
| BPRS total score (mean ± SD) | 45.10 (9.25) | 47.78 (10.55) | 0.019^a^ |
| Unusual Thought Content severity score (mean ± SD) | 2.99 (1.70) | 3.51 (1.67) | 0.003^a,1^ |
| Perceptual Abnormalities severity score (mean ± SD) | 3.07 (1.72) | 3.34 (1.69) | 0.262^a,1^ |
| Disorganized speech severity score (mean ± SD) | 1.84 (1.42) | 2.19 (1.40) | 0.060^a,1^ |
| Impaired cognitive functioning (mean ± SD) | 2.31 (1.13) | 2.63 (1.38) | 0.011^a,1^ |
| Impaired emotional functioning severity score (mean ± SD) | 1.64 (1.41) | 2.16 (1.68) | 0.009^a,1^ |
| Impaired energy severity score (mean ± SD) | 2.62 (1.57) | 3.02 (1.71) | 0.040^a,1^ |
| Impaired motor functioning severity score (mean ± SD) | 0.52 (1.00) | 0.80 (1.25) | 0.151^a,1^ |
| Impaired bodily sensation severity score (mean ± SD) | 0.73 (1.24) | 1.08 (1.55) | 0.185^a,1^ |
| Impaired autonomic functioning severity score (mean ± SD) | 1.48 (1.54) | 1.46 (1.63) | >0.999 ^a,1^ |
| Impaired tolerance to normal stress (mean ± SD) | 2.34 (1.63) | 2.48 (1.85) | >0.999^a,1^ |
| Non-bizarre ideas severity score (mean ± SD) | 3.37 (1.83) | 3.34 (1.56) | >0.999 ^a,1^ |
| Time from baseline assessment to transition (mean ± SD) | | 276.72 (267.33) |  |
| Time between baseline assessment and last follow-up (mean ± SD) | 2740.18 (1111.53) |  |  |
| Enrolled in the context of (%) |  |  |  |
| Cohort study/placebo | 172 (52.92) | 108 (55.38) | 0.650^b^ |
| Intervention treatment | 153 (47.08) | 87 (44.62) |  |
| UHR inclusion criteria (%) |  |  |  |
| BLIPS | 12 (3.69) | 13 (6.67) | 0.151^b^ |
| APS | 214 (65.85) | 131 (67.18) |  |
| APS+BLIPS | 12 (3.69) | 8 (4.10) |  |
| Trait | 44 (13.54) | 12 (6.15) |  |
| BLIPS+Trait | 2 (0.62) | 2 (1.03) |  |
| Trait+APS | 37 (11.38) | 24 (12.31) |  |
| BLIPS+APS+Trait | 4 (1.23) | 4 (2.05) |  |
| NA | 0 (0.0) | 1 (0.51) |  |

**Legend.** APS – attenuated psychotic symptoms; BLIPS – brief limited intermittent psychotic symptoms; BPRS – Brief Psychiatric Rating Scale; GAF – Global Assessment of Functioning; NA – Not applicable; SOFAS – Social and Occupational Functioning Assessment Scale; UHR – ultra-high risk for psychosis; a - Mann-Whitney U test; b – x2 test/Monte Carlo; 1 – After Bonferroni correction

**Table S4.** Proportion of missing data for each variable

|  | % of missing |  |
| --- | --- | --- |
| Age at baseline | 0.4 |  |
| Gender assigned at birth (%) | | 0.0 |
| Time between first symptom and intake at clinical service, days | 7.8 |  |
| GAF score | 35.7 |  |
| SOFAS score | 59.0 |  |
| BPRS total score | 4.4 |  |
| Unusual Thought Content severity score | 4.4 |  |
| Perceptual Abnormalities severity score | 0.0 |  |
| Disorganized speech severity score | 0.2 |  |
| Impaired cognitive functioning | 3.9 |  |
| Impaired emotional functioning severity score | 3.9 |  |
| Impaired energy severity score | 3.5 |  |
| Impaired motor functioning severity score | 4.1 |  |
| Impaired bodily sensation severity score | 4.1 |  |
| Impaired autonomic functioning severity score | 4.3 |  |
| Impaired tolerance to normal stress | 4.3 |  |
| Non-bizarre ideas severity score | 63.4 |  |
| Time between baseline assessment and last follow-up or transition (if after three years) | 0.0 |  |
| Enrolled in the context of (%) |  |  |
| Cohort study/placebo | 0.0 |  |
| Intervention treatment |  |  |
| UHR inclusion criteria | 0.2 |  |

BLIPS – brief limited intermittent psychotic symptoms, APS – attenuated psychotic symptoms, BPRS – Brief Psychiatric Rating Scale; GAF – Global Assessment of Functioning, SOFAS – Social and Occupational Functioning Assessment Scale; UHR – ultra-high risk for psychosis


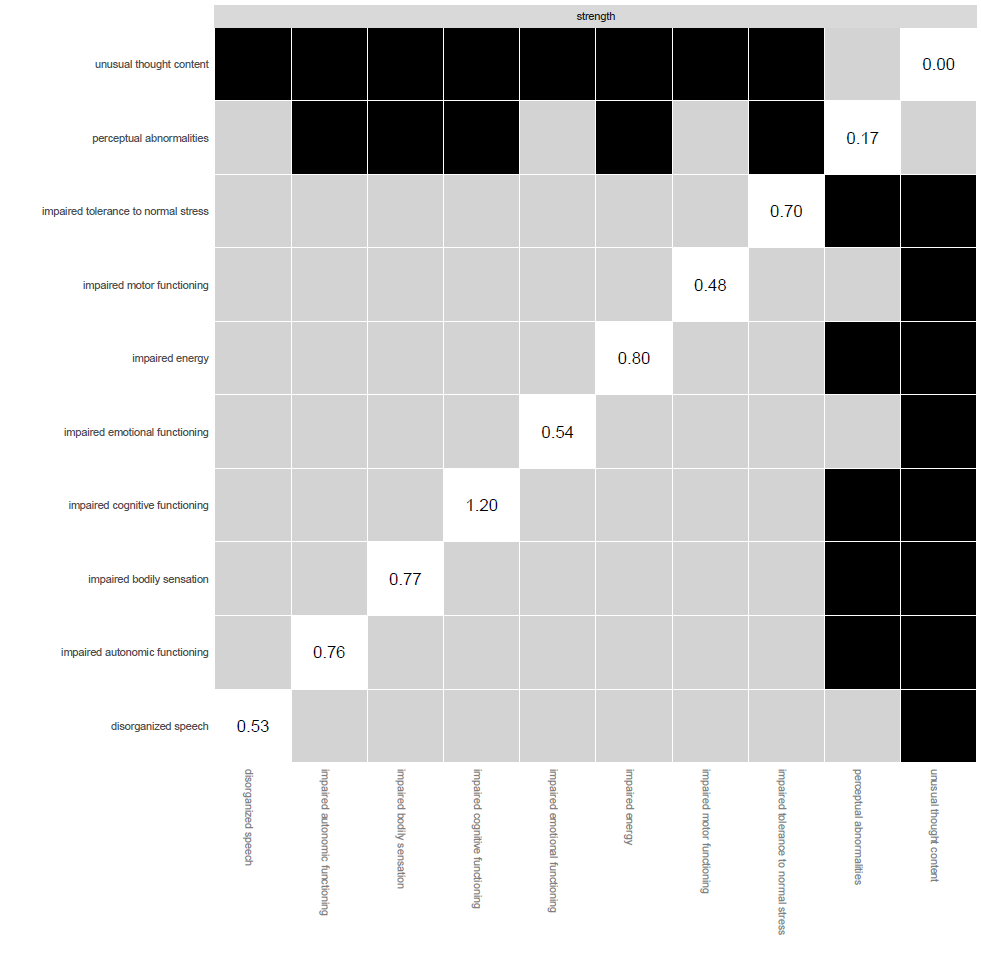


**Figure S1.** Centrality difference test in the network of individuals who transitioned (N = 195). Gray boxes represent nodes that do not show differences, while black boxes indicate nodes that do differ.


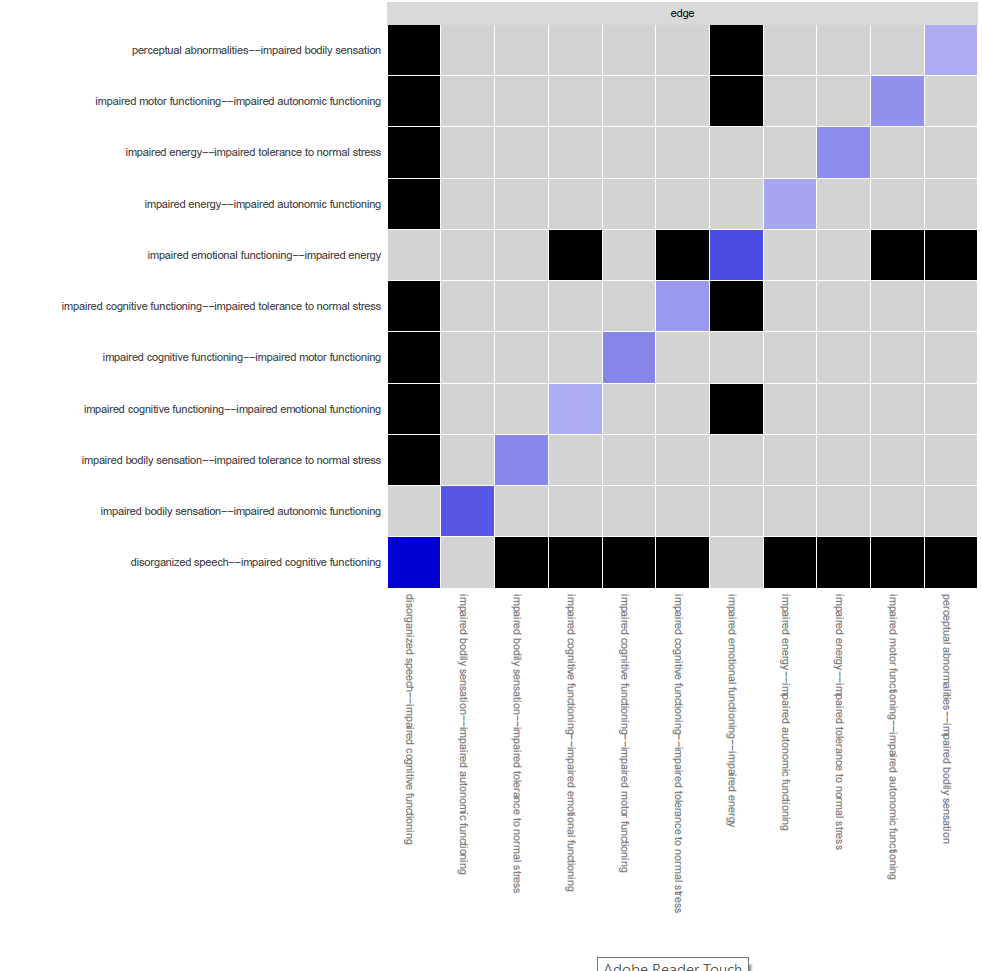


**Figure S2.** Edge weight difference test in the network of individuals who transitioned (N = 195). Gray boxes represent edges that do not show differences, while black boxes indicate edges that do differ.


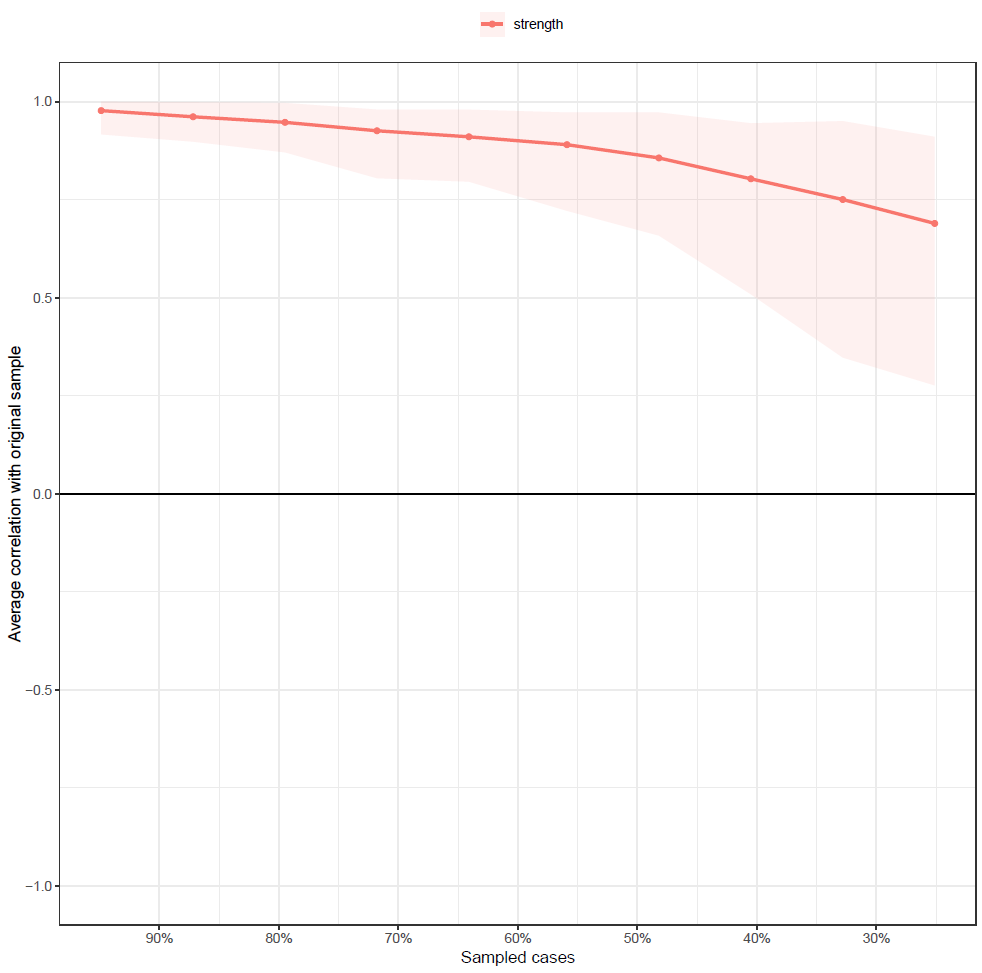


**Figure S3.** Average correlation between the centrality indices of the network subsamples and the original sample in the network of individuals who transitioned (N = 195). Lines indicate means and areas indicate the range from the 2.5th to the 97.5th quantile.


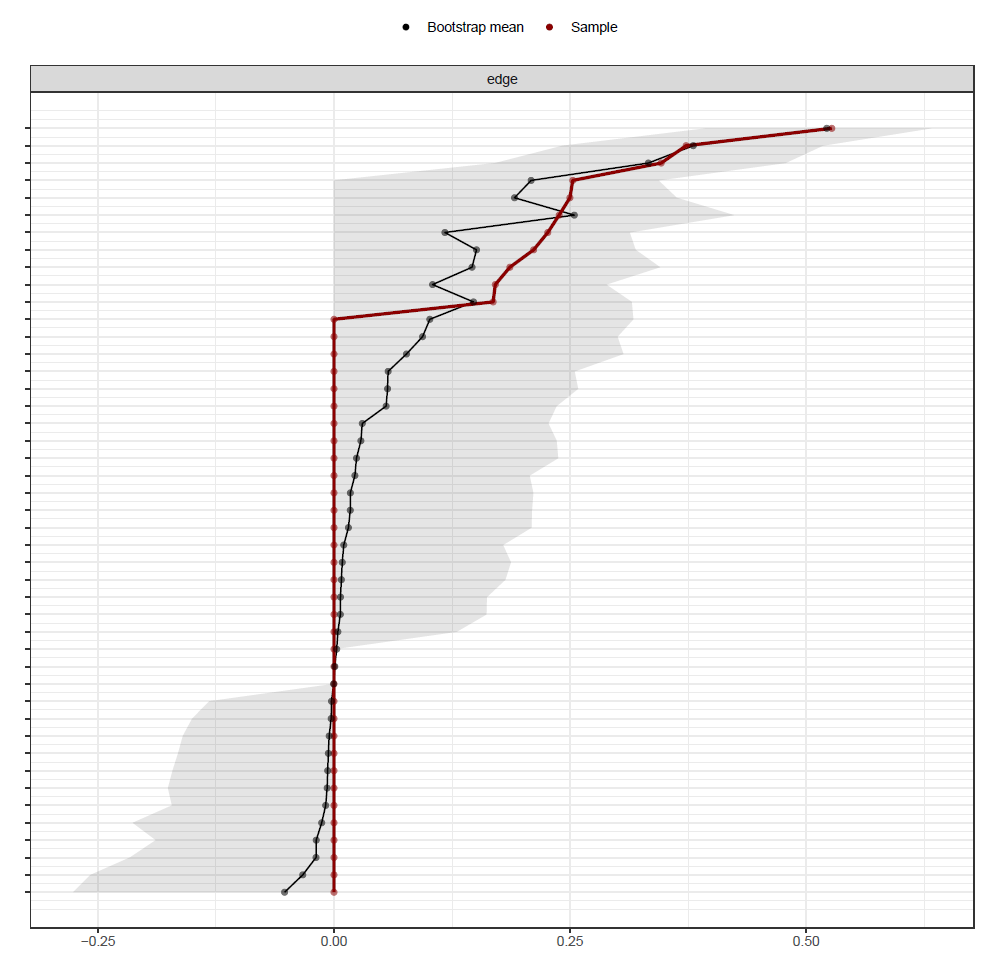


**Figure S4.** Bootstrapped confidence intervals of estimated edge weights for the network of individuals who transitioned (N = 195)


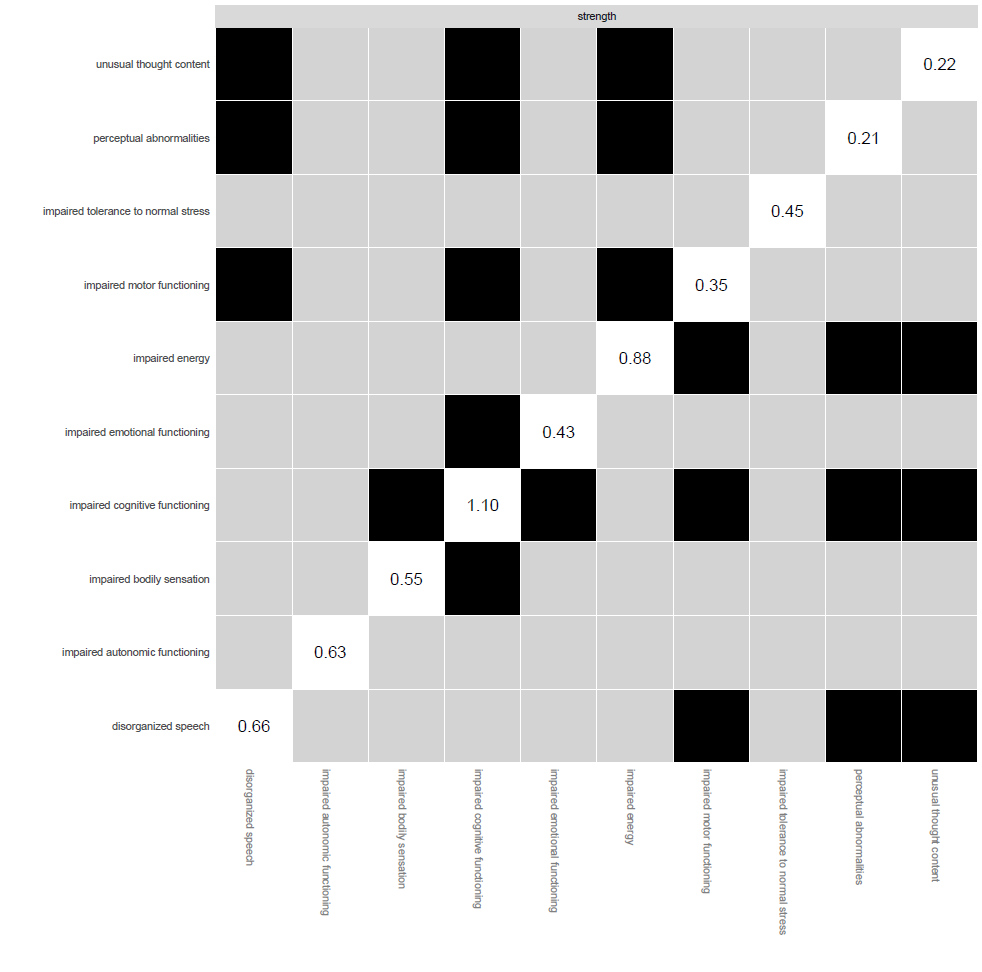


**Figure S5.** Centrality difference test in the network of individuals who did not transition (N = 346). Gray boxes represent nodes that do not show differences, while black boxes indicate nodes that do differ.


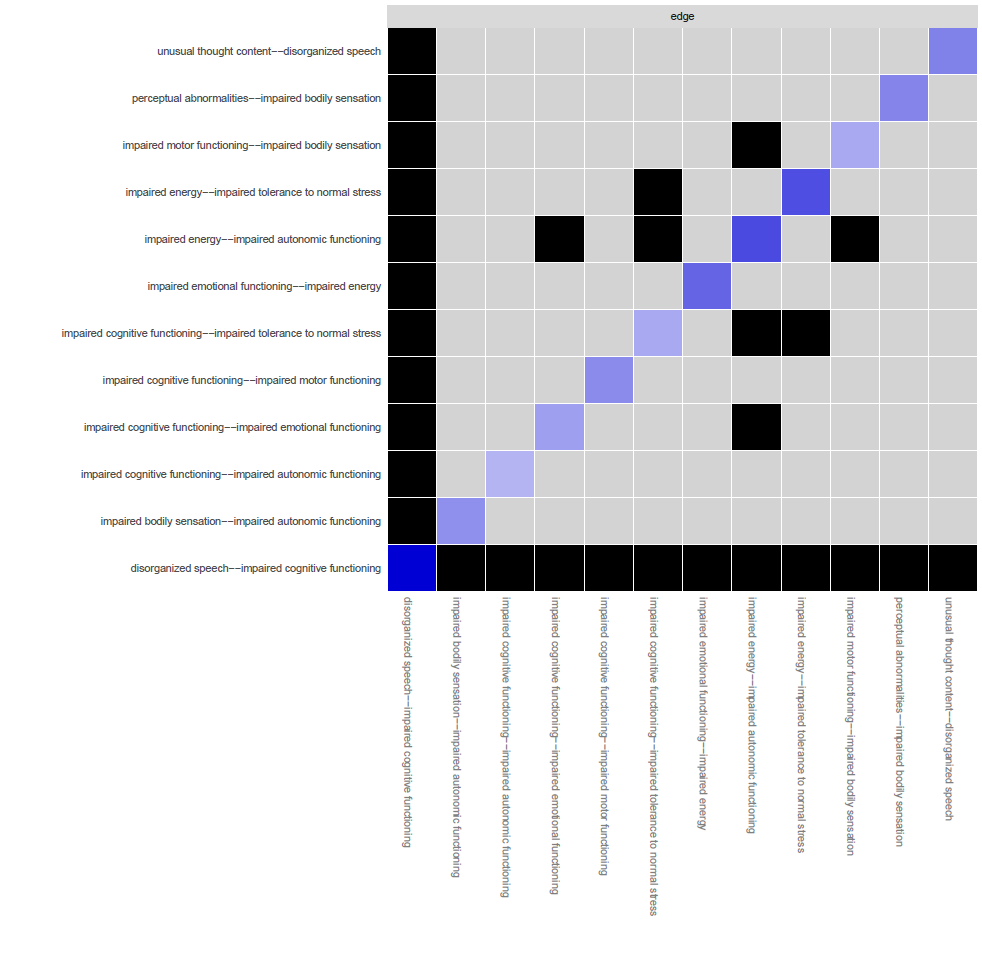


**Figure S6.** Edge weight difference test in the network of individuals who did not transition (N = 346). Gray boxes represent edges that do not show differences, while black boxes indicate edges that do differ.


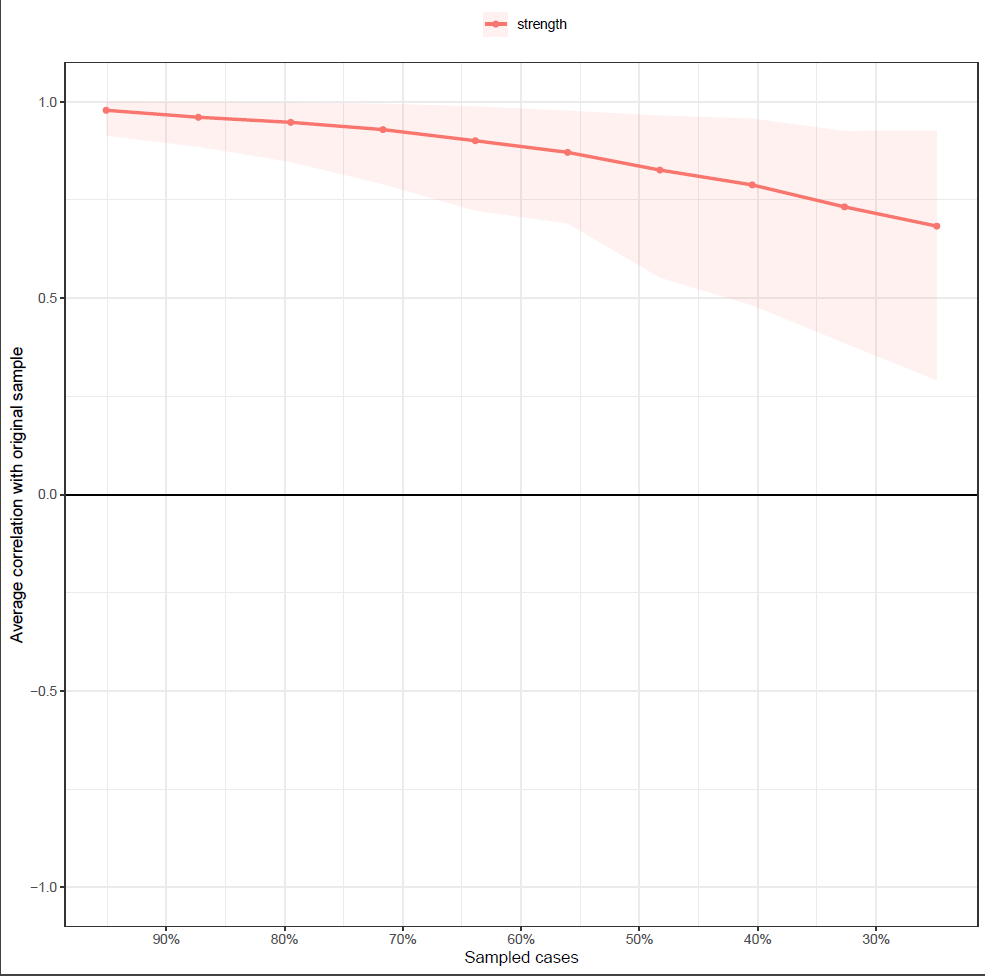


**Figure S7.** Average correlation between the centrality indices of the network subsamples and the original sample in the network of individuals who did not transition (N = 346). Lines indicate means and areas indicate the range from the 2.5th to the 97.5th quantile.


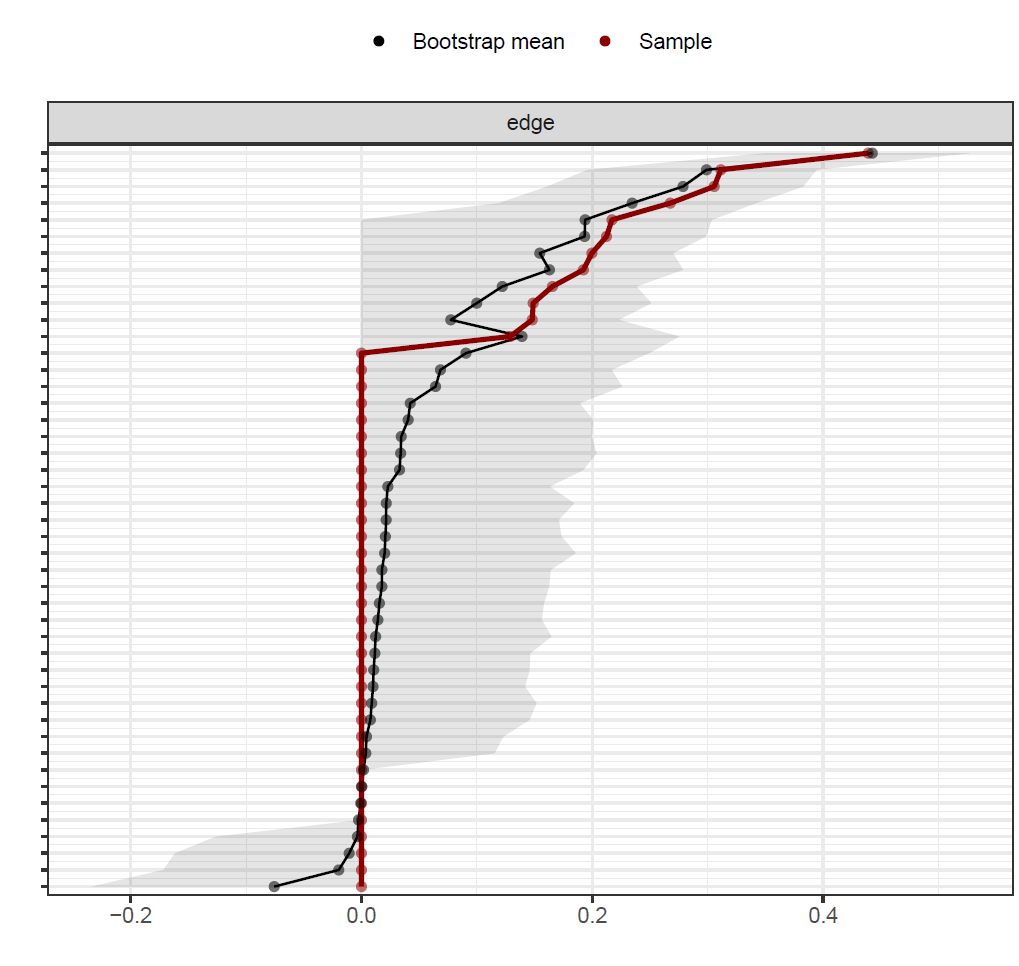
**Figure S8.** Bootstrapped confidence intervals of estimated edge weights for the network of individuals who did not transition (N=346).

.

**TableS5.** Network comparison test to compare the network structures of individuals who transitioned and did not transition

| **Overall strength invariance** | p = .53 |
| --- | --- |
| Network structure invariance | p = .42 |
| Strength (any node)* | p > .05 |

*Non-significant without applying Bonferroni correction


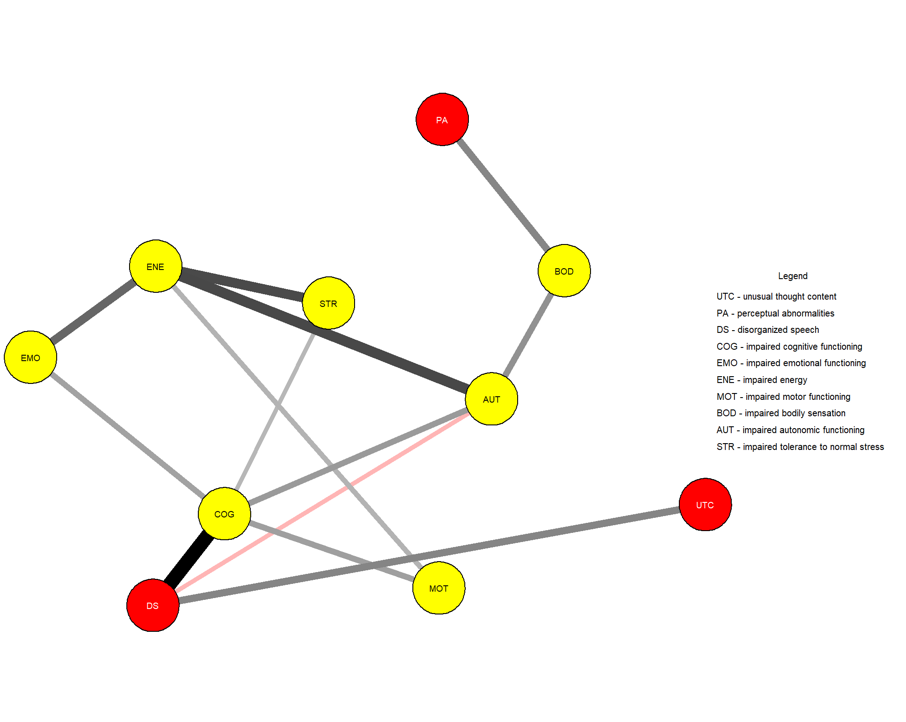


**Figure S9.** Network structure of individuals who did not transition excluding those who transitioned after three years (N = 325). The associations are either positive (colored black) or negative (colored red), with thicker lines representing stronger associations


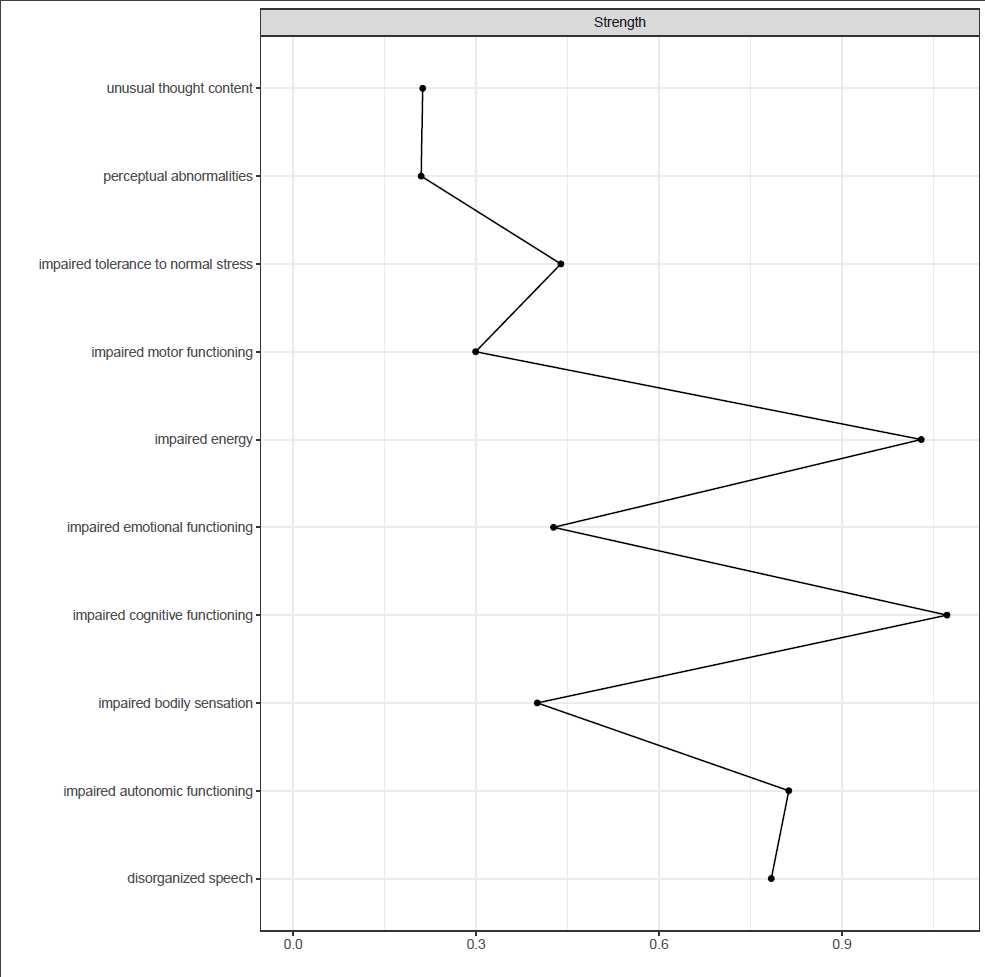


**Figure S10.** Centrality index (strength) in individuals who did not transition excluding those who transitioned after three years (N = 325), shown as standardized z-scores


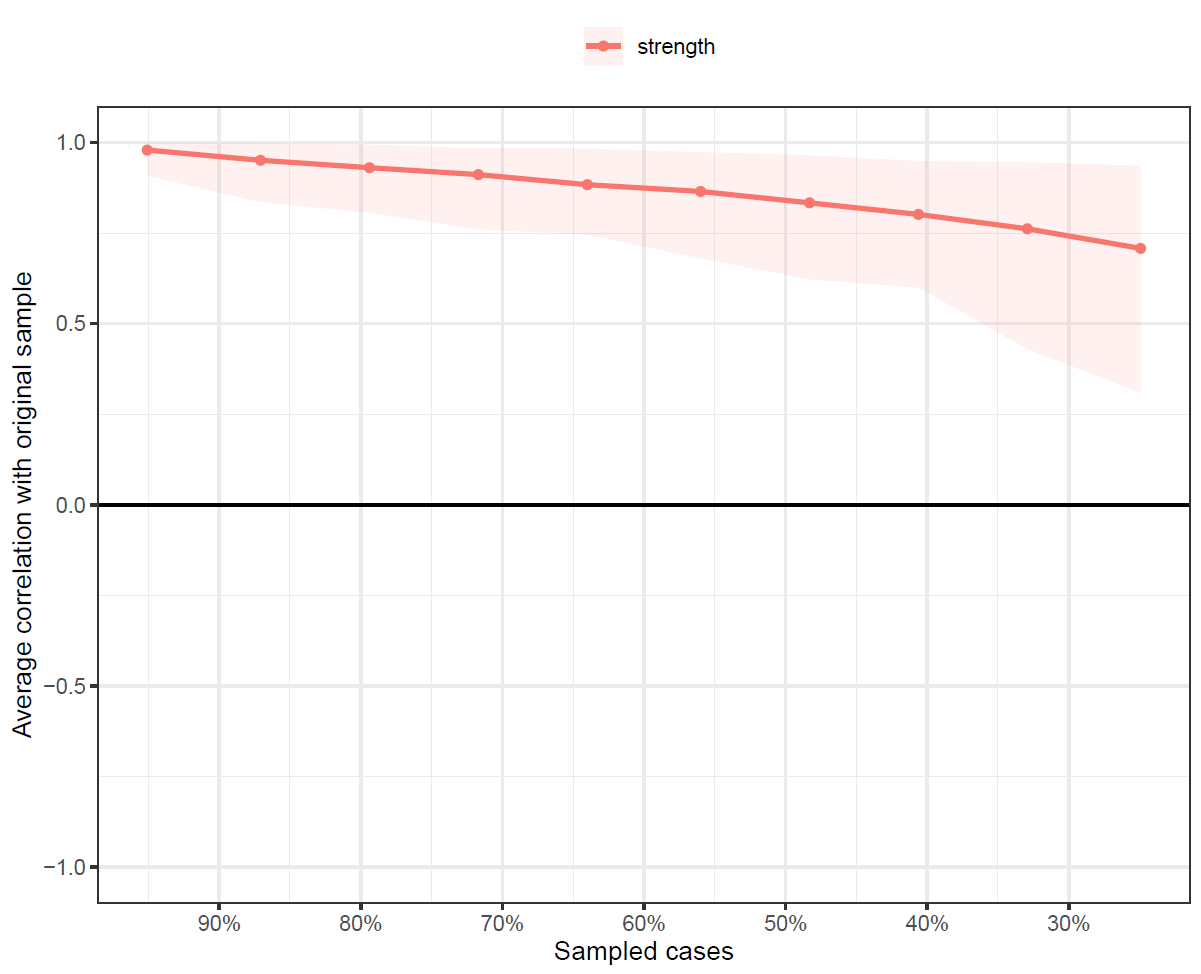


**Figure S11.** Average correlation between the centrality indices of the network subsamples and the original sample in individuals who did not transition excluding those who transitioned after three years (N = 325). CS = .44. Lines indicate means and areas indicate the range from the 2.5th to the 97.5th quantile.


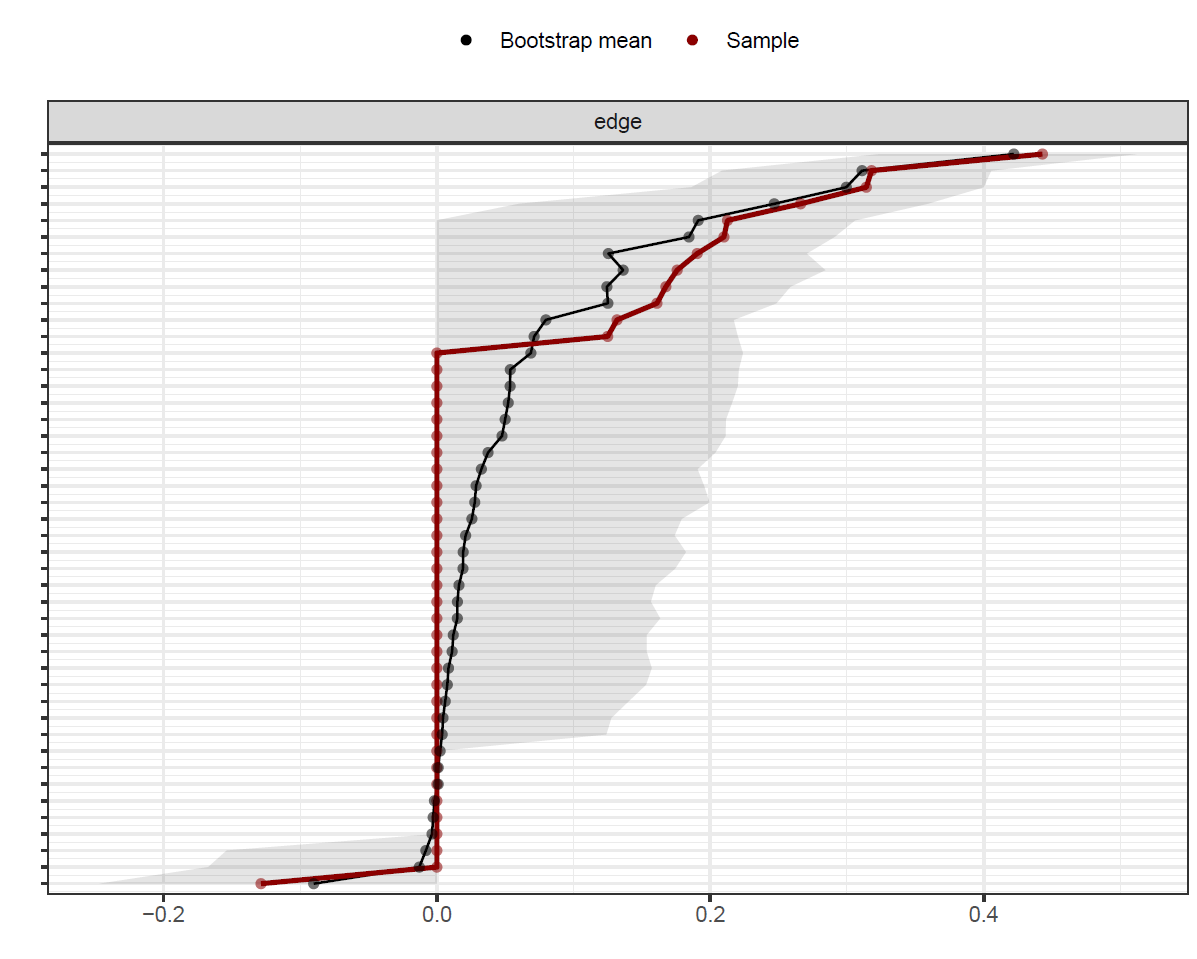


**Figure S12.** Bootstrapped confidence intervals of estimated edge weights for the network of individuals who did not transition within three years excluding those who transitioned after three years (N = 325).

**TableS6.** Network comparison test to compare the network structures of individuals who transitioned and who did not transition within three years, excluding those who transitioned after three years

| **Overall strength invariance** | p = .742 |
| --- | --- |
| Network structure invariance | p = .385 |
| Strength (any node)* | p > .05 |

*Non-significant without applying Bonferroni correction


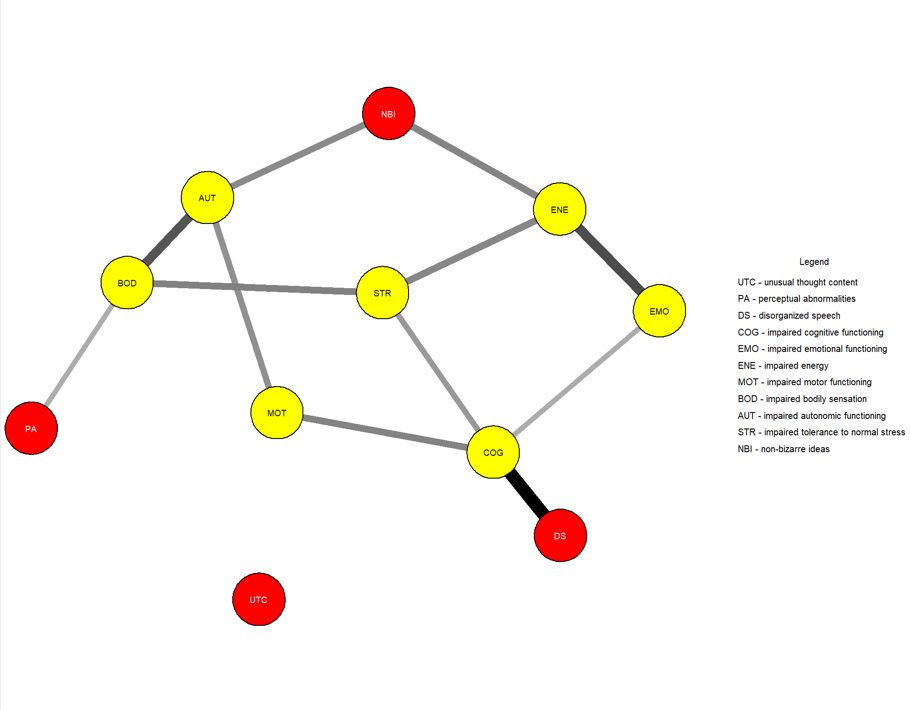


**Figure S13.** Network structure of individuals who transitioned including the node “non-bizarre ideas” (N = 195). The associations are either positive (colored black) or negative (colored red), with thicker lines representing stronger associations


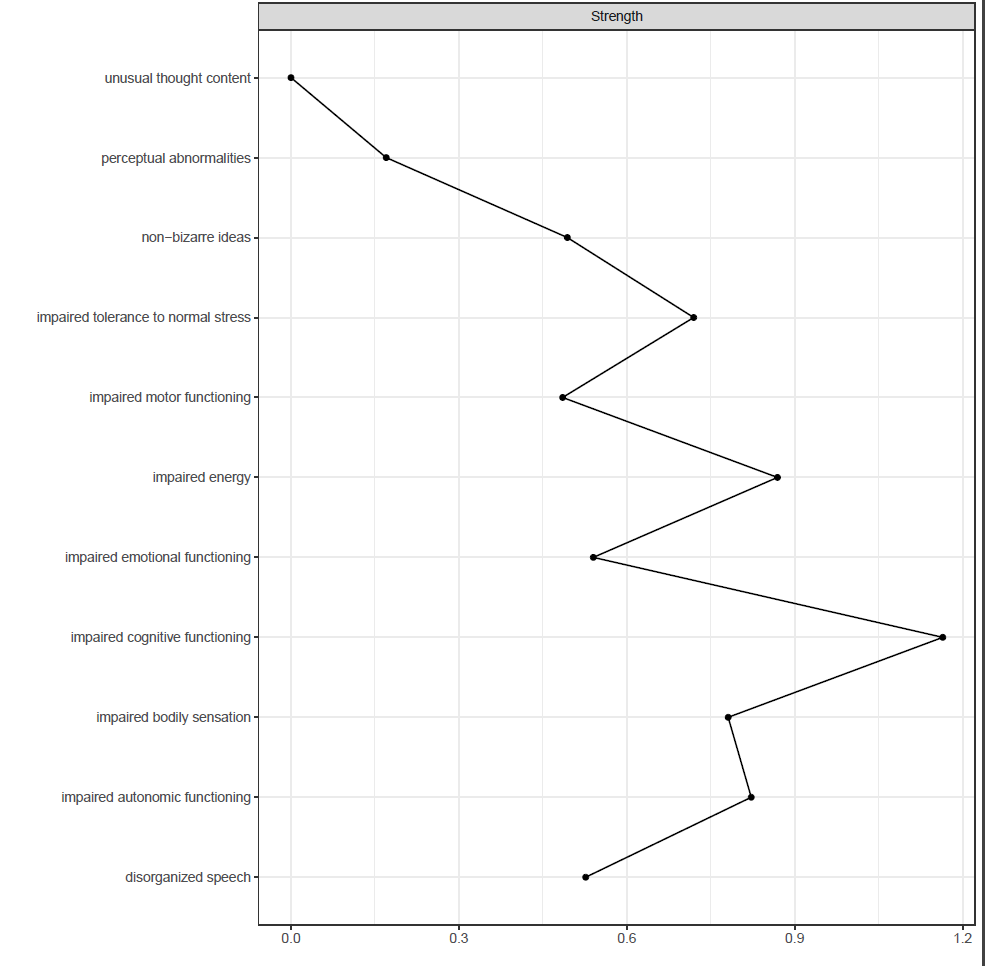


**Figure S14.** Centrality index (strength) in the network of individuals who transitioned including “non-bizarre ideas” (N = 195), shown as standardized z-scores


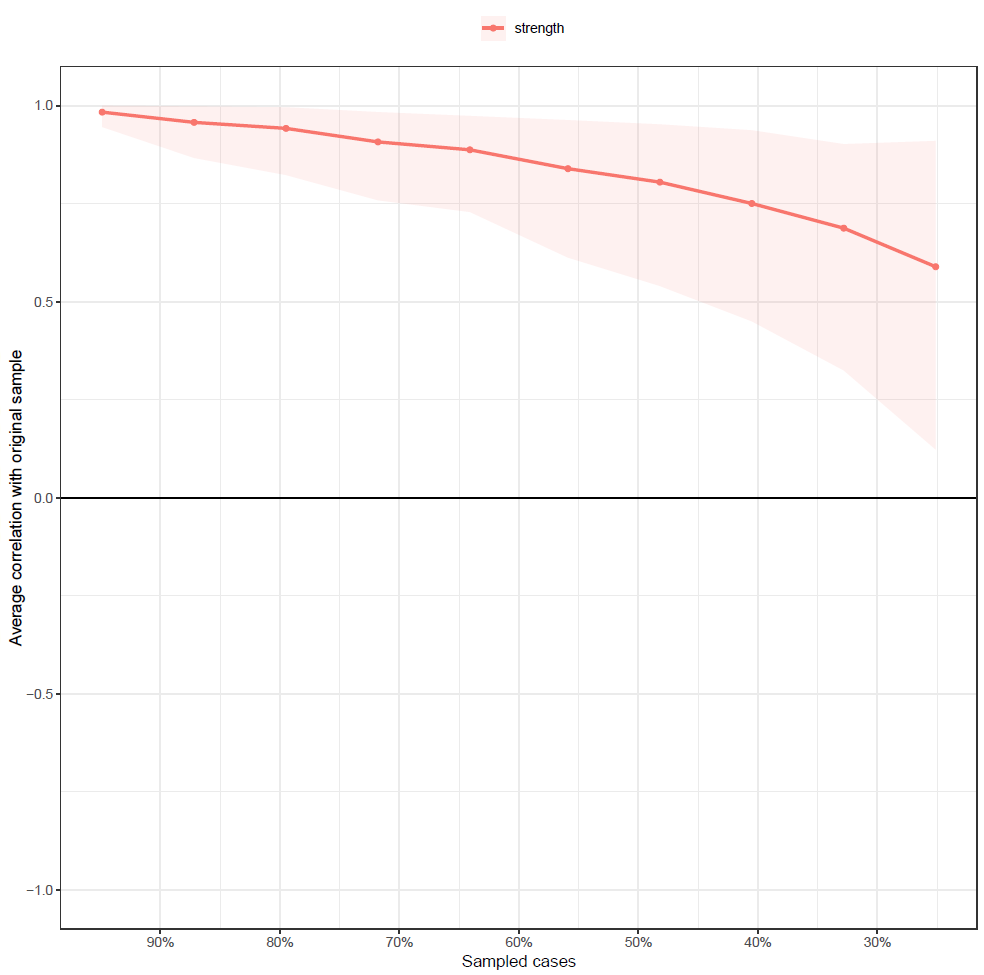


**Figure S15.** Average correlation between the centrality indices of the network subsamples and the original sample in the network of individuals who transitioned including “non bizarre ideas” (N = 195). CS = .36. Lines indicate means and areas indicate the range from the 2.5th to the 97.5th quantile.


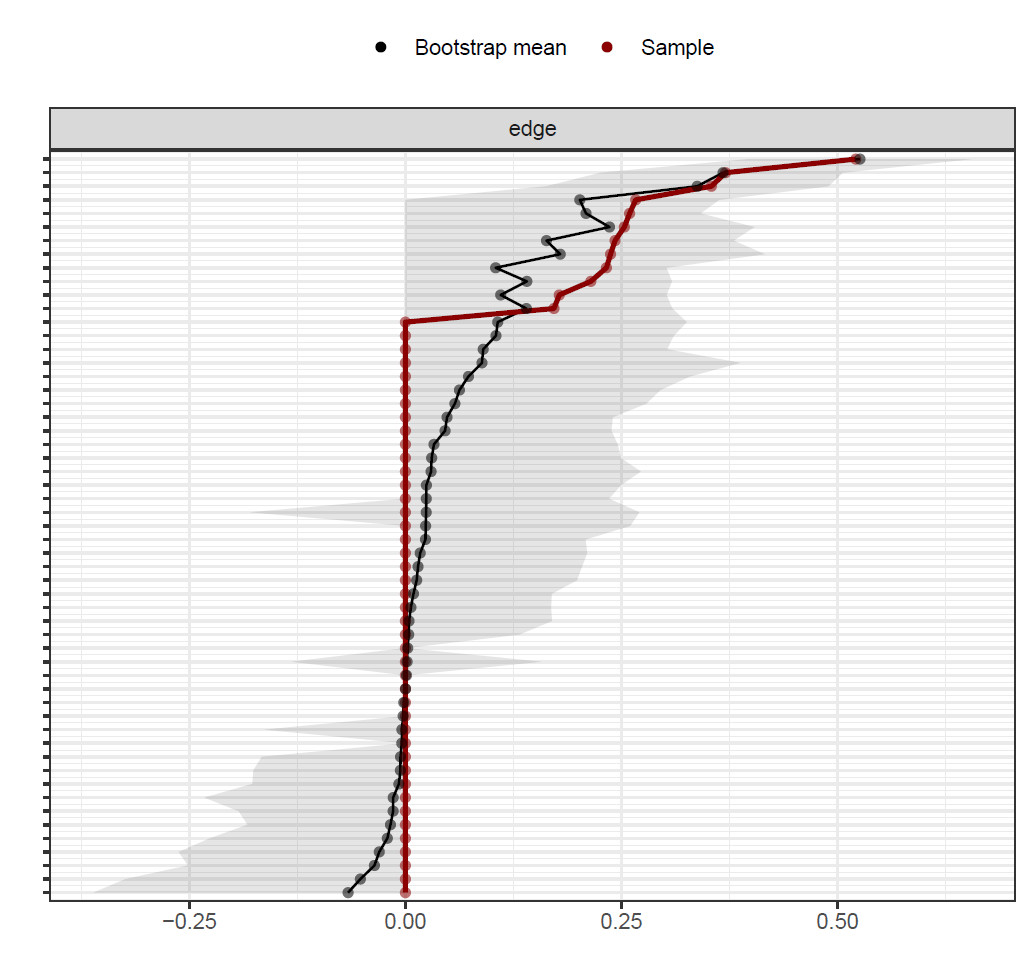


**Figure S16.** Bootstrapped confidence intervals of estimated edge weights for the network of individuals who transitioned including “non-bizarre ideas” (N = 195).


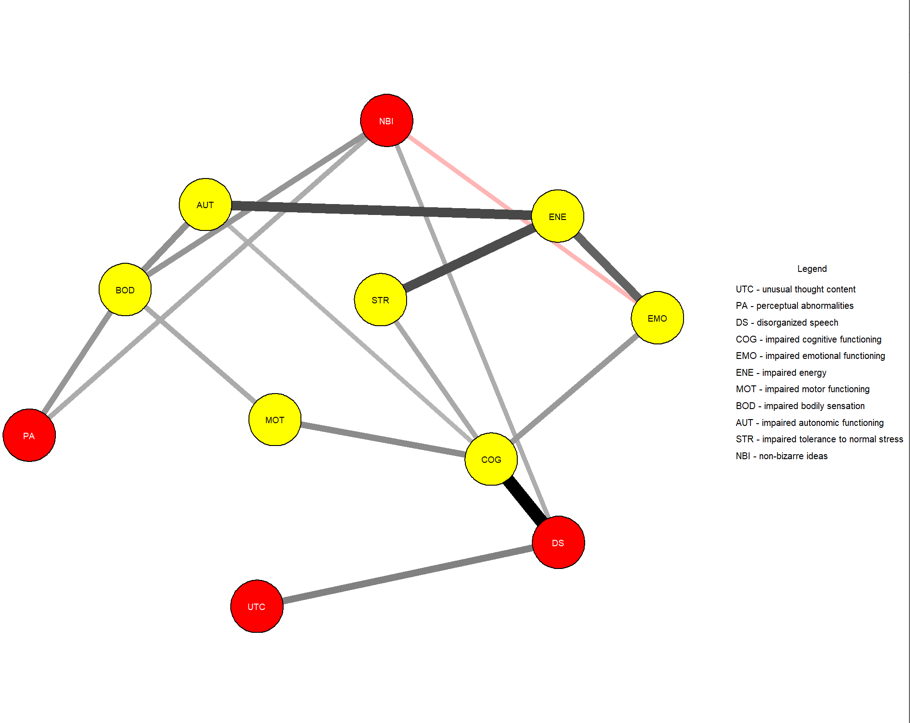


**Figure S17.** Network structure of individuals who did not transition including the node “non-bizarre ideas” (N = 346). The associations are either positive (colored black) or negative (colored red), with thicker lines representing stronger associations


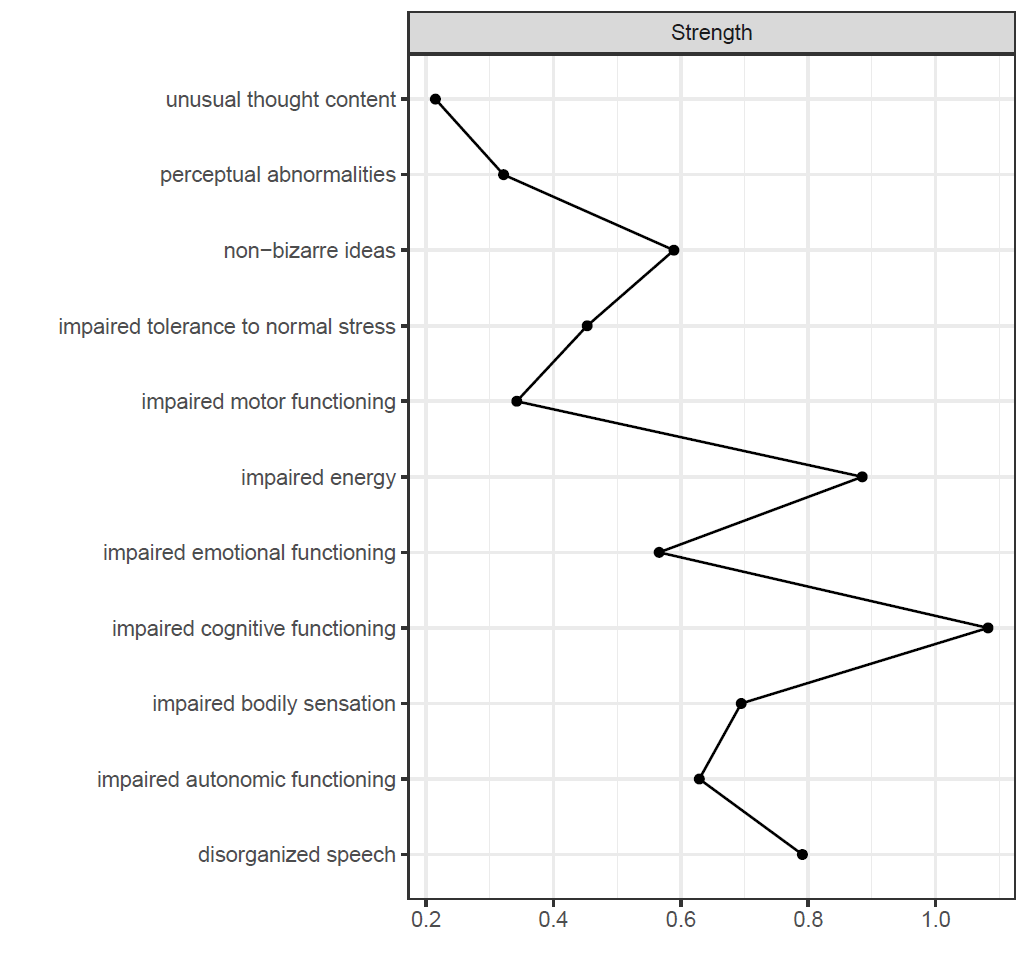


**Figure S18.** Centrality index (strength) in the network of individuals who did not transition including “non-bizarre ideas” (N = 346), shown as standardized z-scores.


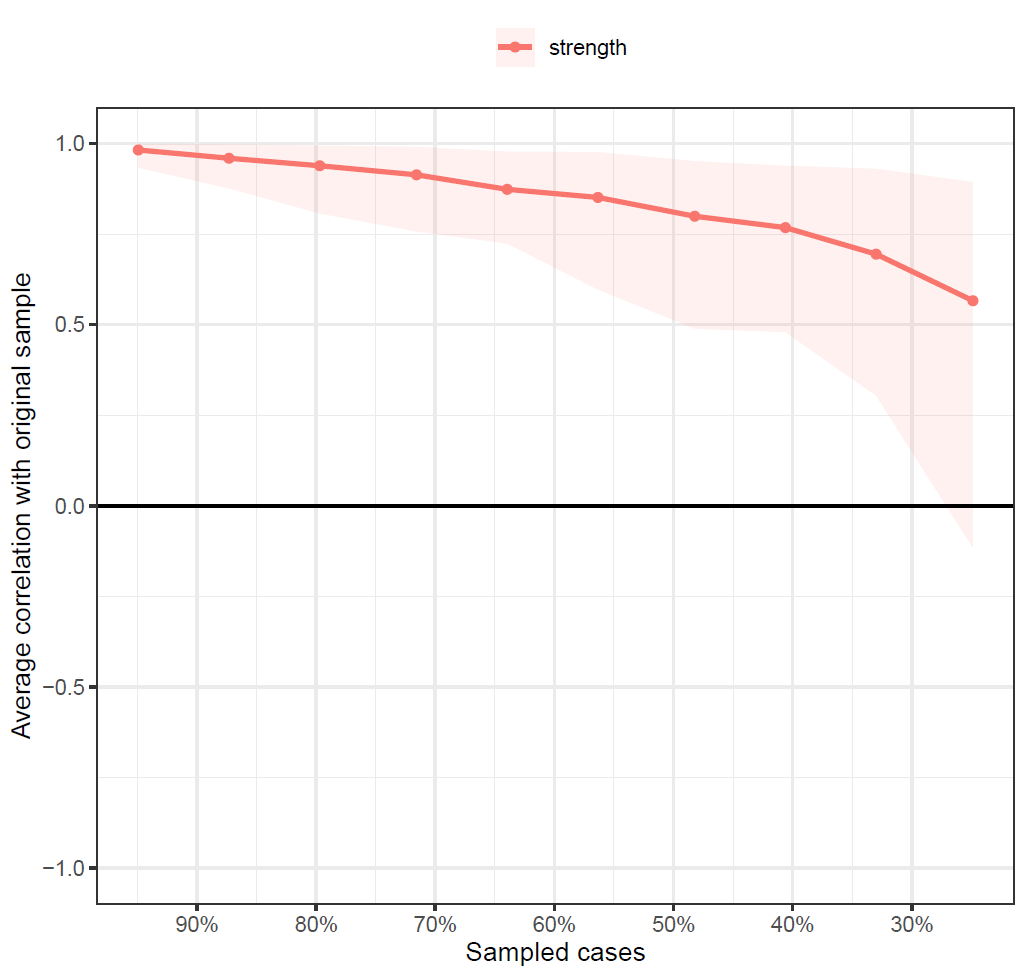


**Figure S19.** Average correlation between the centrality indices of the network subsamples and the original sample in the network of individuals who did not transition including “non bizarre ideas” (N = 346). CS = .21. Lines indicate means and areas indicate the range from the 2.5th to the 97.5th quantile.


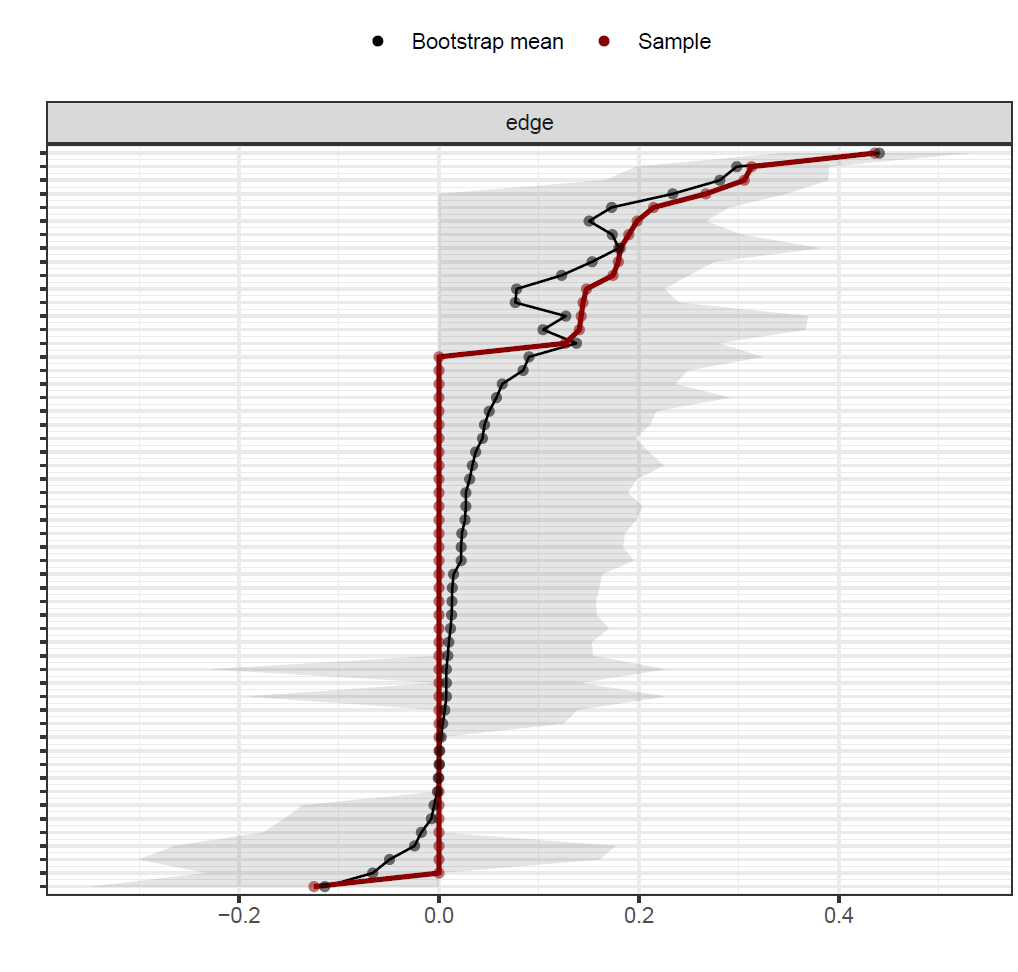


**Figure S20.** Bootstrapped confidence intervals of estimated edge weights for the network of individuals who did not transition including “non-bizarre ideas” (N = 346).

**TableS7.** Network comparison test to compare the network of individuals who transitioned and did not transition, both including “non-bizarre ideas”

| **Overall strength invariance** | p = .993 |
| --- | --- |
| Network structure invariance | p = .210 |
| Strength (any node)* | p > .05 |

*Non-significant without applying Bonferroni correction


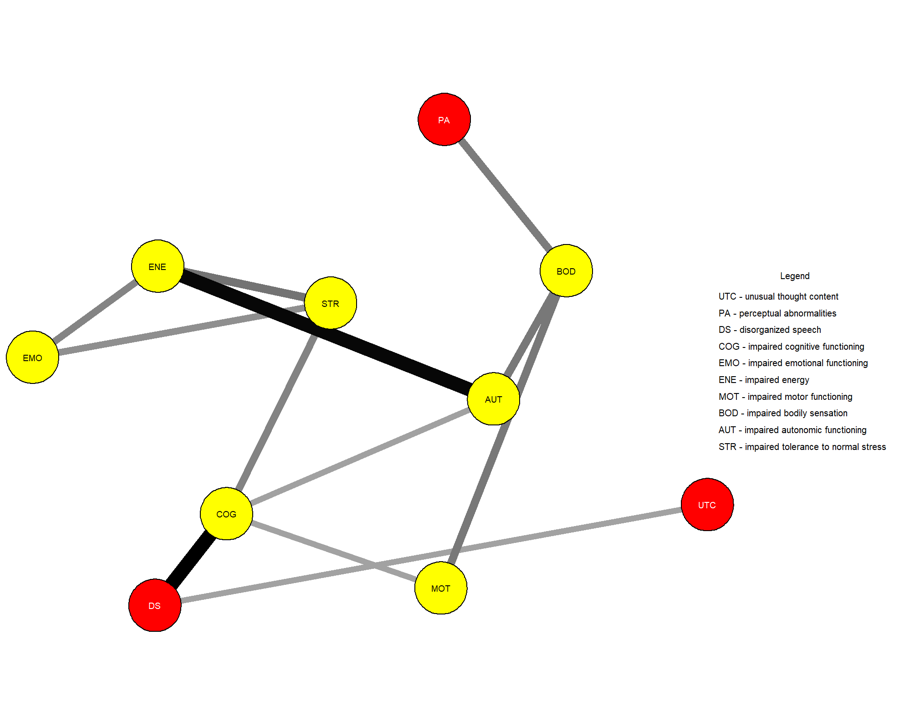


**Figure S21.** Network structure of the matched sub-group of individuals who did not transition (N = 195). The associations are either positive (colored black) or negative (colored red), with thicker lines representing stronger associations


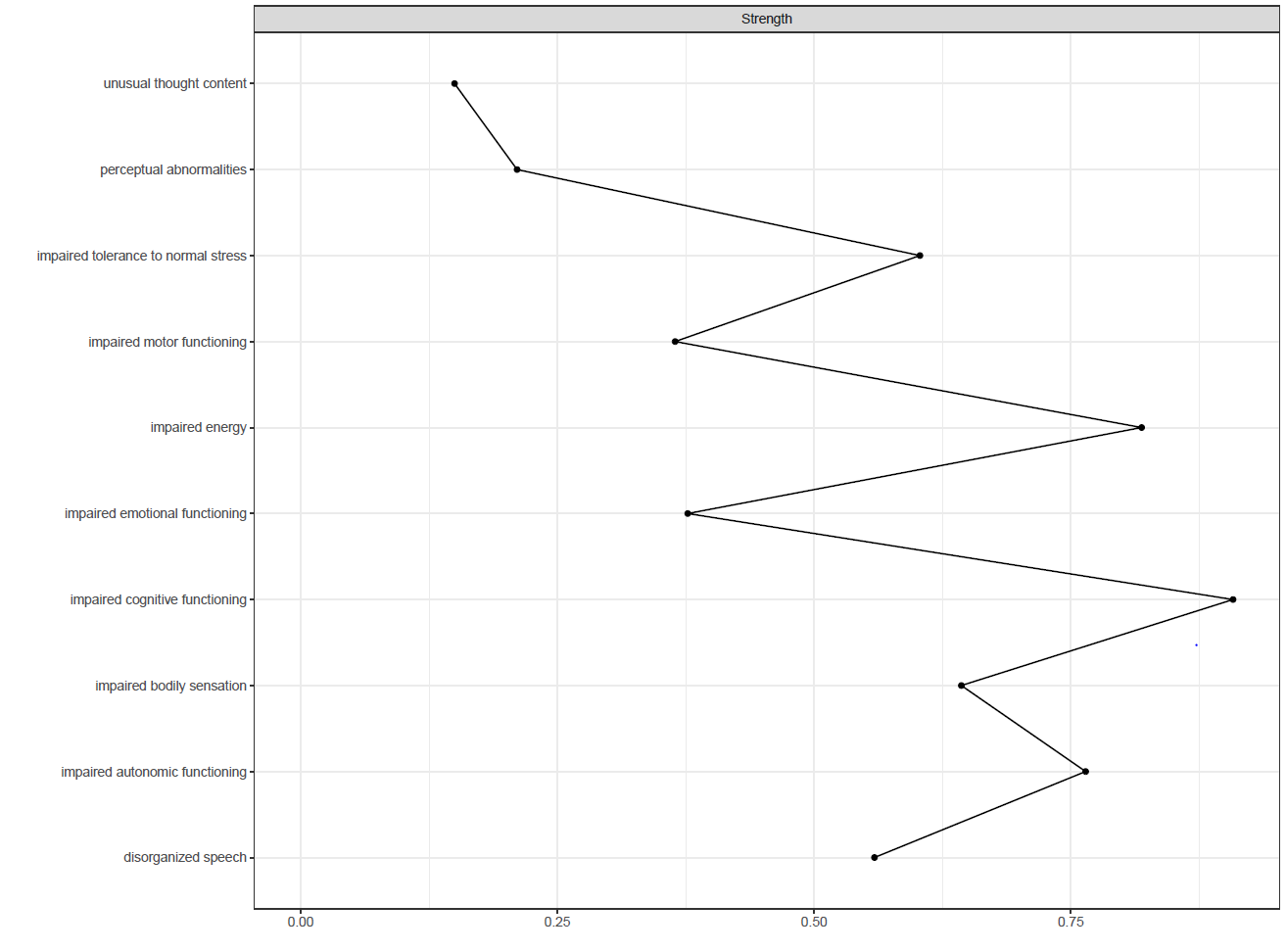


**Figure S22.** Centrality index (strength) in the matched sub-group of individuals who did not transition (N = 195), shown as standardized z-scores.


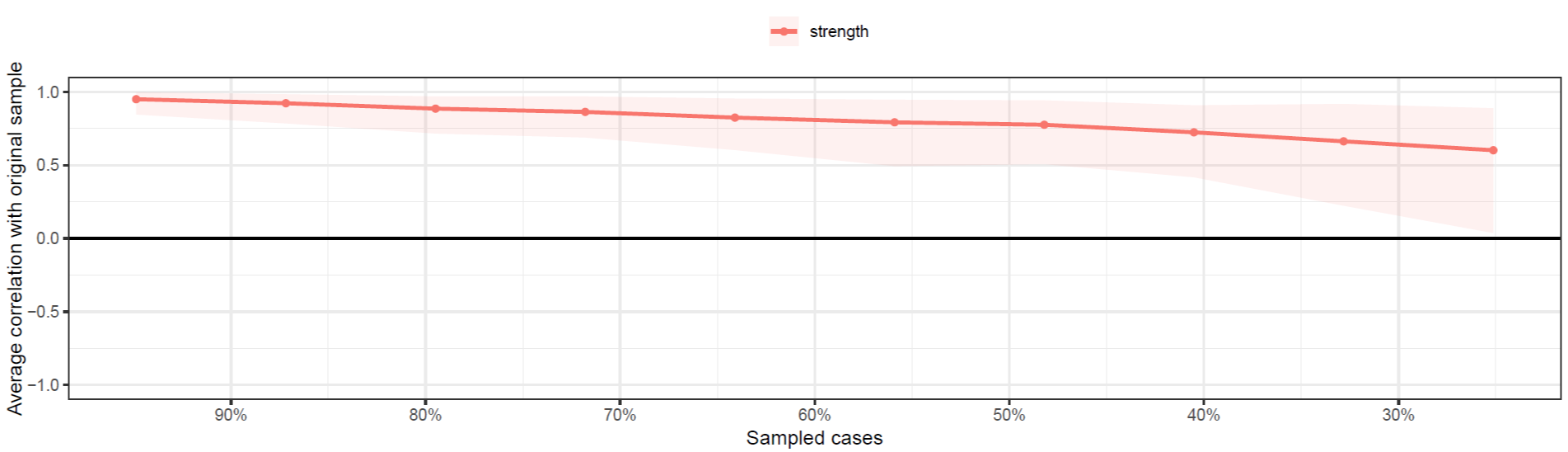


**Figure S23.** Average correlation between the centrality indices of the network subsamples and the original sample in the network of the matched sub-group of individuals who did not transition (N = 195). CS = .28. Lines indicate means and areas indicate the range from the 2.5th to the 97.5th quantile.


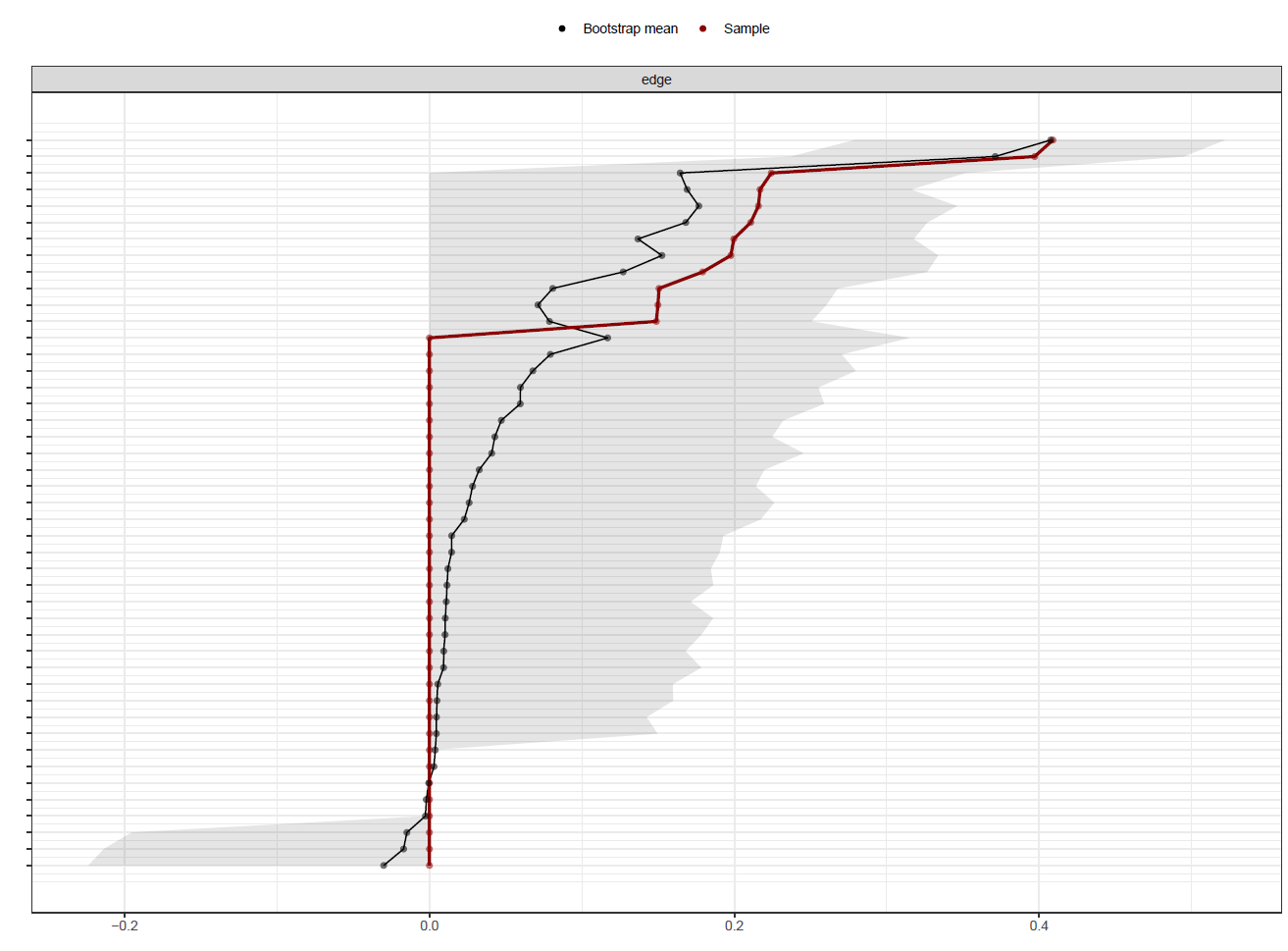


**Figure S24.** Bootstrapped confidence intervals of estimated edge weights for the network of the matched sub-group of individuals who did not transition

**TableS8.** Network comparison test to compare the network of individuals transitioning and matched sub-group of individuals who did not transition

| **Overall strength invariance** | p = .507 |
| --- | --- |
| Network structure invariance | p = .750 |
| Strength (any node)* | p > .05 |

*Non-significant without applying Bonferroni correction


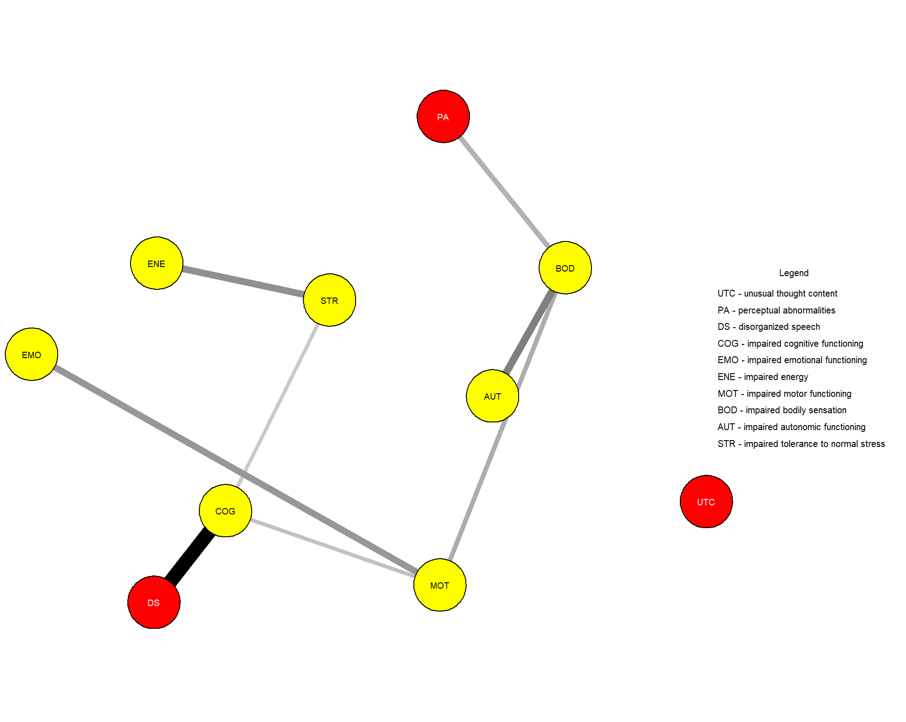


**Figure S25.** Network structure of individuals who transitioned using non-harmonized CAARMS versions (0-4 scores) (N = 62). The associations are either positive (colored black) or negative (colored red), with thicker lines representing stronger associations


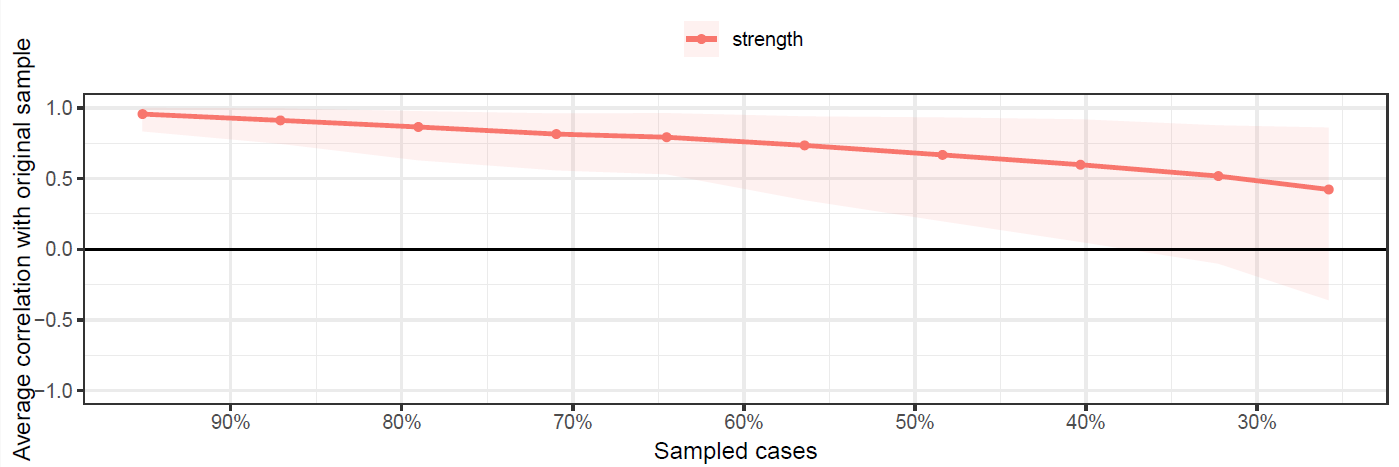


**Figure S26.** Average correlation between the centrality indices of the network subsamples and the original sample in the network of individuals who transitioned using non-harmonized CAARMS versions (0-4 scores). (N = 62). CS = .21. Lines indicate means and areas indicate the range from the 2.5th to the 97.5th quantile.


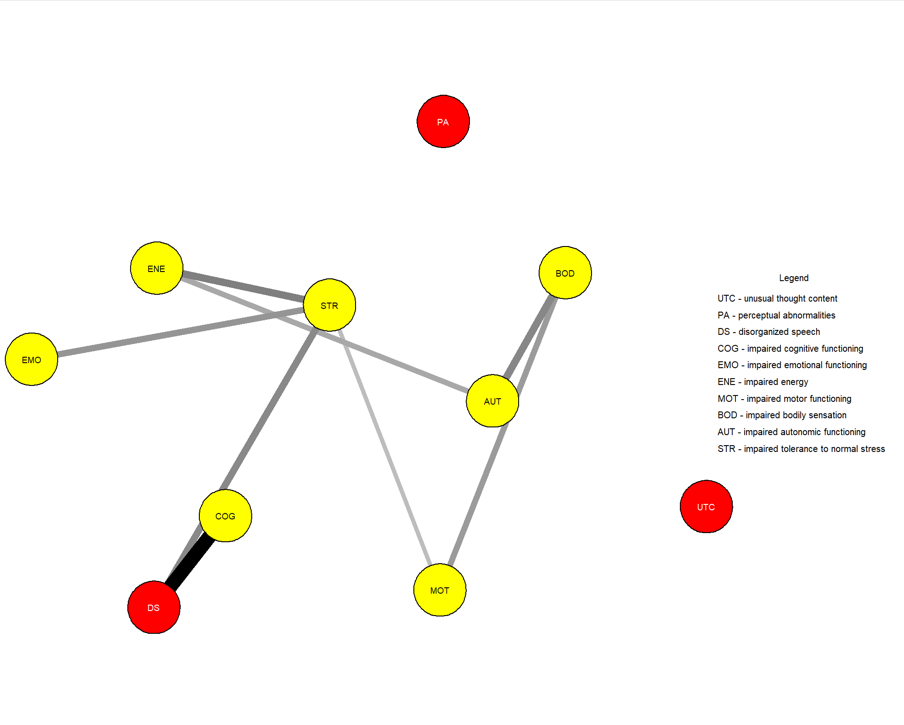


**Figure S27.** Network structure of individuals who did not transition using non-harmonized CAARMS versions (0-4 scores). (N = 95). The associations are either positive (colored black) or negative (colored red), with thicker lines representing stronger associations


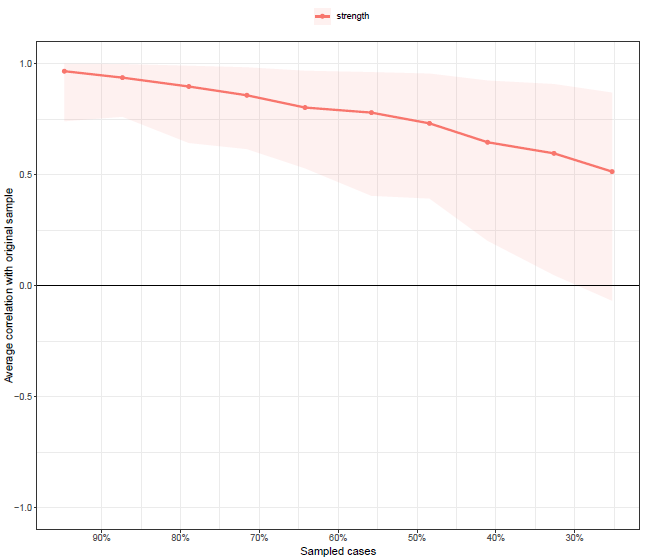


**Figure S28.** Average correlation between the centrality indices of the network subsamples and the original sample in the network of individuals who did not transition using non-harmonized CAARMS versions (0-4 scores) (N = 95). CS = .21. Lines indicate means and areas indicate the range from the 2.5th to the 97.5th quantile.

**TableS9.** Network comparison test to compare the network of individuals transitioning and individuals who did not transition using non-harmonized CAARMS versions (0-4 scores)

| **Overall strength invariance** | p = .766 |
| --- | --- |
| Network structure invariance | p = .721 |
| Strength (any node)* | p > .05 |

*Non-significant without applying Bonferroni correction


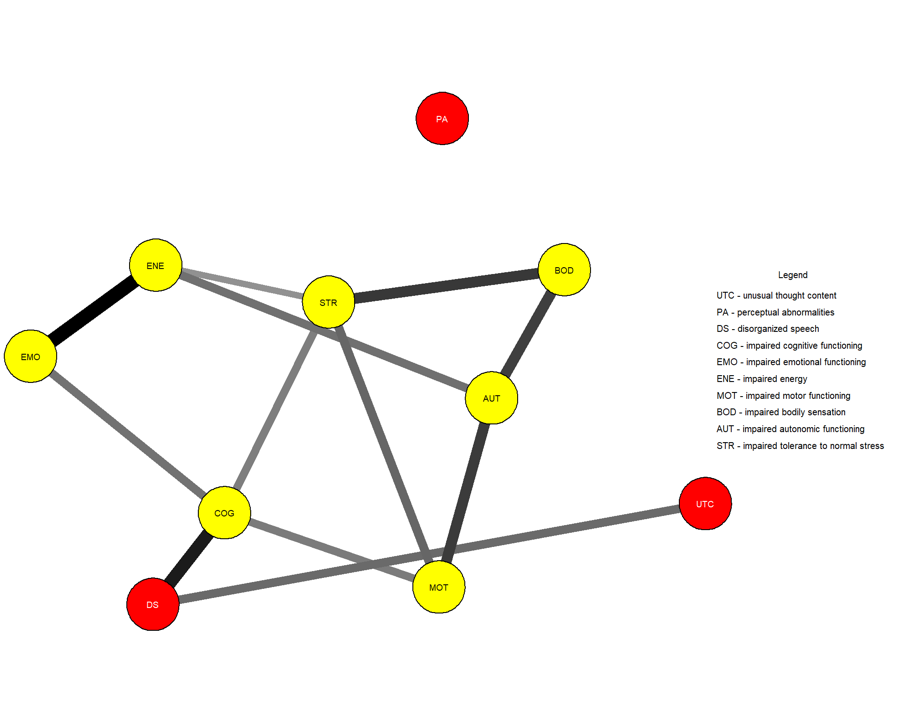


**Figure S29.** Network structure of individuals who transitioned using non-harmonized CAARMS versions (0-6 scores) (N = 133). The associations are either positive (colored black) or negative (colored red), with thicker lines representing stronger associations


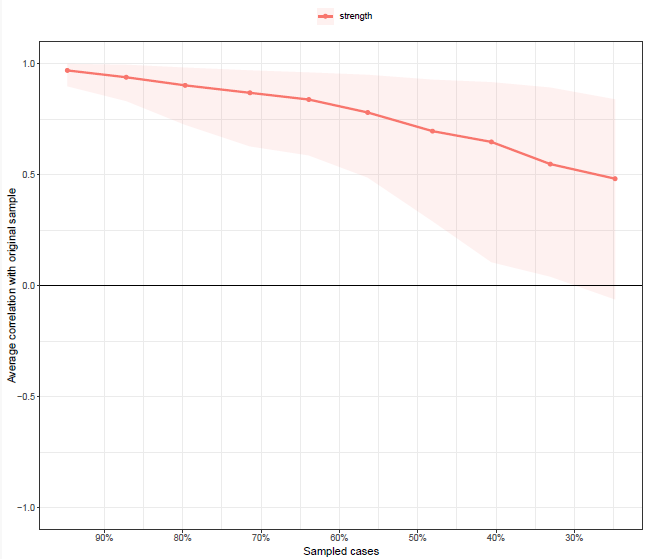


**Figure S30.** Average correlation between the centrality indices of the network subsamples and the original sample in the network of individuals who transitioned using non-harmonized CAARMS versions (0-6 scores) (N = 133) CS = .20. Lines indicate means and areas indicate the range from the 2.5th to the 97.5th quantile.


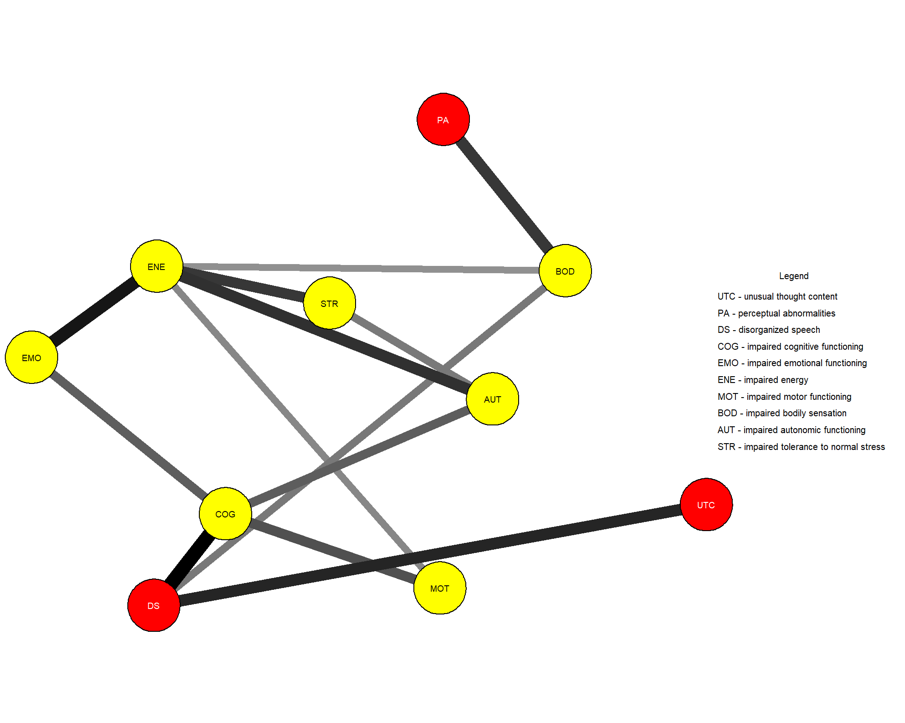


**Figure S31.** Network structure of individuals who did not transition using non-harmonized CAARMS versions (0-6 scores). (N = 251). The associations are either positive (colored black) or negative (colored red), with thicker lines representing stronger associations.


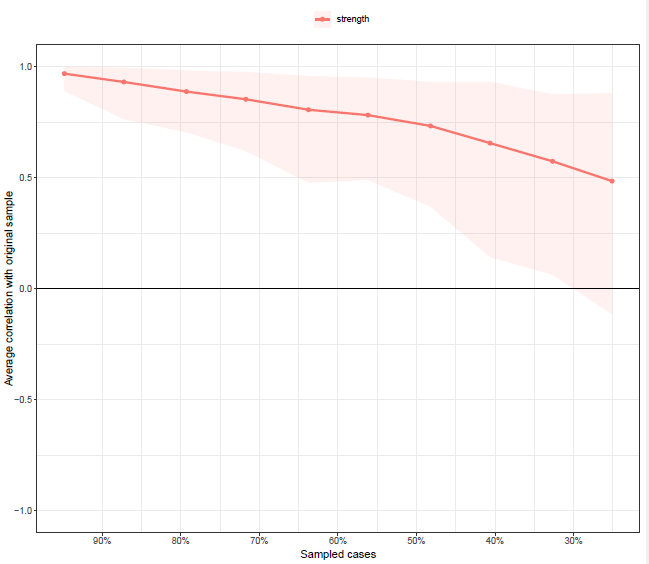


**Figure S32.** Average correlation between the centrality indices of the network subsamples and the original sample in the network of individuals who did not transition using non-harmonized CAARMS versions (0-6 scores) (N = 251) CS = .21. Lines indicate means and areas indicate the range from the 2.5th to the 97.5th quantile.

**TableS10.** Network comparison test to compare the network of individuals transitioning and individuals who did not transition using non-harmonized CAARMS versions (0-6 scores)

| **Overall strength invariance** | p = .518 |
| --- | --- |
| Network structure invariance | p = .471 |
| Strength (any node)* | p > .05 |

*Non-significant without applying Bonferroni correction

**Table S11.** Socio-demographic and clinical characteristics at baseline of excluded UHR individuals and included UHR individuals did not transition to psychosis within three years

|  | Included individuals who did not transition within three years | Excluded individuals who did not transition | p-Value |
| --- | --- | --- | --- |
| Number of subjects | 346 | 698 |  |
| Age at baseline (mean ± SD) | 18.51 (3.47) | 18.33 (3.68) | 0.318^a^ |
| Gender assigned at birth (%) |  |  |  |
| Male | 153 (44.22) | 310 (44.41) | 1.000^b^ |
| Female | 193 (55.78) | 388 (55.59) |  |
| Time between first symptom and intake at clinical service, days (mean ± SD) | 404.77 (778.66) | 771.83 (1108.76) | <0.001^a^ |
| GAF score (mean ± SD) | 59.88 (10.71) | 58.95 (11.25) | 0.485^a^ |
| SOFAS score (mean ± SD) | 54.85 (11.74) | 55.62 (12.00) | 0.486^a^ |
| BPRS total score (mean ± SD) | 45.38 (9.31) | 43.22 (9.04) | <0.001^a^ |
| Unusual thought content severity score (mean ± SD) | 3.02 (1.67) | 2.71 (1.87) | 0.406^a,1^ |
| Perceptual abnormalities severity score (mean ± SD) | 3.09 (1.72) | 3.37 (1.43) | 0.851^a,1^ |
| Disorganized speech severity score (mean ± SD) | 1.87 (1.43) | 1.51 (1.34) | 0.003^a,1^ |
| Impaired cognitive functioning (mean ± SD) | 2.35 (1.13) | 2.48 (1.24) | 0.086^a,1^ |
| Impaired emotional functioning severity score (mean ± SD) | 1.65 (1.44) | 2.20 (1.57) | <0.001^a,1^ |
| Impaired energy severity score (mean ± SD) | 2.61 (1.57) | 3.27 (1.52) | <0.001^a,1^ |
| Impaired motor functioning severity score (mean ± SD) | 0.55 (1.03) | 0.59 (1.01) | 1.000^a,1^ |
| Impaired bodily sensation severity score (mean ± SD) | 0.75 (1.26) | 0.73 (1.30) | 1.000^a,1^ |
| Impaired autonomic functioning severity score (mean ± SD) | 1.48 (1.54) | 1.50 (1.54) | 1.000^a,1^ |
| Impaired tolerance to normal stress (mean ± SD) | 2.33 (1.66) | 2.38 (1.88) | 1.000^a,1^ |
| Non-bizarre ideas severity score (mean ± SD) | 3.40 (1.83) | 3.17 (1.62) | 0.699^a,1^ |
| Time between baseline assessment and last follow-up (mean ± SD) | 2687.47 (1106.50) | 444.83 (293.38) |  |
| Enrolled in the context of (%) |  |  |  |
| Cohort study/placebo | 181 (52.31) | 283 (40.54) | <0.001^b^ |
| Intervention treatment | 165 (47.69) | 415 (59.46) |  |
| UHR inclusion criteria (%) |  |  | <0.001^b^ |
| BLIPS | 12 (3.47) | 6 (0.86) |  |
| APS | 226 (65.32) | 549 (78.65) |  |
| APS+BLIPS | 15 (4.34) | 11 (1.58) |  |
| Trait | 47 (13.58) | 38 (5.44) |  |
| BLIPS+Trait | 2 (0.58) | 2 (0.29) |  |
| Trait+APS | 40 (11.56) | 79 (11.32) |  |
| BLIPS+APS+Trait | 4 (1.16) | 7 (1.00) |  |
| NA | 0 (0.0) | 6 (0.86) |  |

**Legend.** APS – attenuated psychotic symptoms; BLIPS – brief limited intermittent psychotic symptoms; BPRS – Brief Psychiatric Rating Scale; GAF – Global Assessment of Functioning; NA – Not applicable; SOFAS – Social and Occupational Functioning Assessment Scale; UHR – ultra-high risk for psychosis; a - Mann-Whitney U test; b – x^2^ test/Monte Carlo; 1 – After Bonferroni correction


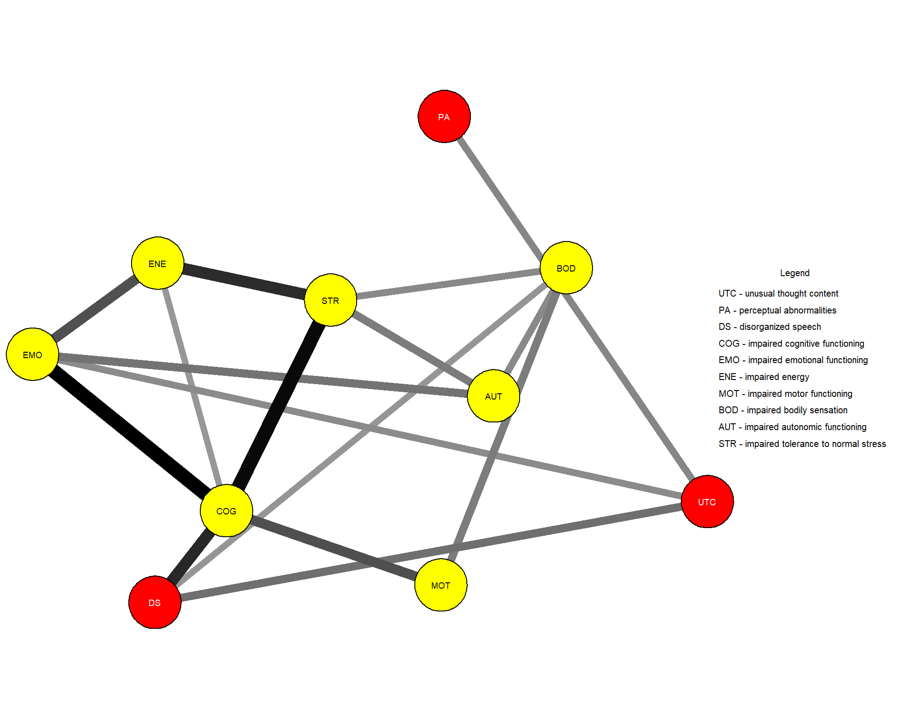


**Figure S33.** Network structure of excluded individuals who did not transition (N = 698). The associations are either positive (colored black) or negative (colored red), with thicker lines representing stronger associations


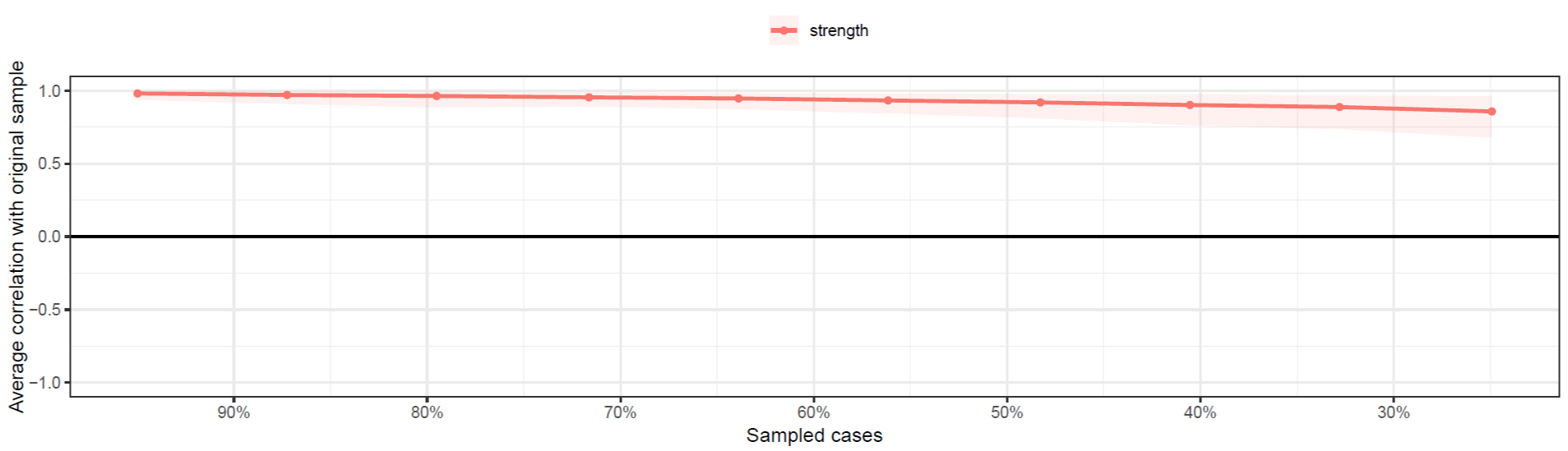


**Figure S34.** Average correlation between the centrality indices of the network subsamples and the original sample in the network of excluded individuals who did not transition (N = 698). CS = .75. Lines indicate means and areas indicate the range from the 2.5th to the 97.5th quantile.

**Table S12.** Network comparison test to compare the network of excluded individuals and included individuals who did not transition to psychosis within three years

| **Overall strength invariance** | p = .717 |
| --- | --- |
| Network structure invariance | p = .005 |
| Impaired tolerance to normal stress | p = .025* |
| Strength (other nodes) | P > .05 |
| Strength of individual edge weights |  |
| Disorganized speech -- Impaired cognitive functioning | p = .004* |
| Perceptual Abnormalities – Impaired bodily sensation | P = .014* |
| Impaired energy – Impaired autonomic functioning | p < .001 (p=0.045 after Bonferroni correction) |
| Other edges | p > .05 |

*Not significant after Bonferroni correction

**Supplementary references**

[1] Rickwood D, Paraskakis M, Quin D, Hobbs N, Ryall V, Trethowan J, et al. Australia’s innovation in youth mental health care: The headspace centre model. Early Interv Psychiatry 2019;13:159–66. https://doi.org/https://doi.org/10.1111/eip.12740.

[2] Amminger GP, Nelson B, Markulev C, Yuen HP, Schäfer MR, Berger M, et al. The NEURAPRO biomarker analysis: long-chain omega-3 fatty acids improve 6-month and 12-month outcomes in youths at ultra-high risk for psychosis. Biol Psychiatry 2020;87:243–52.

[3] Hartmann S, Dwyer D, Cavve B, Byrne EM, Scott I, Gao C, et al. Development and temporal validation of a clinical prediction model of transition to psychosis in individuals at ultra‐high risk in the UHR 1000+ cohort. World Psychiatry 2024;23:400–10.

[4] Yung AR, Phillips LJ, Yuen HP, Francey SM, McFarlane CA, Hallgren M, et al. Psychosis prediction: 12-month follow up of a high-risk (“prodromal”) group. Schizophr Res 2003;60:21–32.

[5] McGorry PD, Yung AR, Phillips LJ, Yuen HP, Francey S, Cosgrave EM, et al. Randomized controlled trial of interventions designed to reduce the risk of progression to first-episode psychosis in a clinical sample with subthreshold symptoms. Arch Gen Psychiatry 2002;59:921–8.

[6] Thompson KN, Phillips LJ, Komesaroff P, Yuen HP, Wood SJ, Pantelis C, et al. Stress and HPA-axis functioning in young people at ultra high risk for psychosis. J Psychiatr Res 2007;41:561–9.

[7] E Berger G, J Wood S, Ross M, A Hamer C, Mark Wellard R, Pell G, et al. Neuroprotective effects of low-dose lithium in individuals at ultra-high risk for psychosis. A longitudinal MRI/MRS study. Curr Pharm Des 2012;18:570–5.

[8] Yung AR, Phillips LJ, Nelson B, Francey SM, PanYuen H, Simmons MB, et al. Randomized controlled trial of interventions for young people at ultra high risk for psychosis: 6-month analysis. J Clin Psychiatry 2010;71:16654.

[9] Phillips LJ, Nelson B, Yuen HP, Francey SM, Simmons M, Stanford C, et al. Randomized controlled trial of interventions for young people at ultra-high risk of psychosis: study design and baseline characteristics. Australian & New Zealand Journal of Psychiatry 2009;43:818–29.

[10] Nelson B, Thompson A, Yung AR. Basic self-disturbance predicts psychosis onset in the ultra high risk for psychosis “prodromal” population. Schizophr Bull 2012;38:1277–87.

[11] McGorry PD, Nelson B, Markulev C, Yuen HP, Schäfer MR, Mossaheb N, et al. Effect of ω-3 polyunsaturated fatty acids in young people at ultrahigh risk for psychotic disorders: the NEURAPRO randomized clinical trial. JAMA Psychiatry 2017;74:19–27.

[12] (EU-GEI) EN of NN studying G-EI in S. Identifying gene-environment interactions in schizophrenia: contemporary challenges for integrated, large-scale investigations. Schizophr Bull 2014;40:729–36.

[13] Nelson B, Lavoie S, Li E, Sass LA, Koren D, McGorry PD, et al. The neurophenomenology of early psychosis: an integrative empirical study. Conscious Cogn 2020;77:102845.

[14] Hartmann JA, Nelson B, Spooner R, Paul Amminger G, Chanen A, Davey CG, et al. Broad clinical high‐risk mental state (CHARMS): Methodology of a cohort study validating criteria for pluripotent risk. Early Interv Psychiatry 2019;13:379–86.

[15] Nelson B, Amminger GP, Yuen HP, Wallis N, J. Kerr M, Dixon L, et al. Staged treatment in early psychosis: a sequential multiple assignment randomised trial of interventions for ultra high risk of psychosis patients. Early Interv Psychiatry 2018;12:292–306.

[16] Tognin S, van Hell HH, Merritt K, Winter-van Rossum I, Bossong MG, Kempton MJ, et al. Towards precision medicine in psychosis: benefits and challenges of multimodal multicenter studies—PSYSCAN: translating neuroimaging findings from research into clinical practice. Schizophr Bull 2020;46:432–41.

[17] Bayer JMM, Spark J, Krcmar M, Formica M, Gwyther K, Srivastava A, et al. The SPEAK study rationale and design: A linguistic corpus-based approach to understanding thought disorder. Schizophr Res 2023;259:80–7.
